# Supercritical Solvent Impregnation of Poly(lactic acid) (PLA)-Based Films: Effect of Poly(3-hydroxybutyrate) (PHB) and Poly(butylene succinate) (PBS) on Loading Capacity, Optical Properties and Release Kinetics of Mango Leaf Extract

**DOI:** 10.3390/polym18111377

**Published:** 2026-06-01

**Authors:** Ludisbel León-Marcos, Antonio Montes, Diego Valor, Ignacio García-Casas, Clara Pereyra

**Affiliations:** Department of Chemical Engineering and Food Technology, Faculty of Sciences, University of Cadiz, International Excellence Agrifood Campus (CeiA3), Wine and Agrifood Research Institute (IVAGRO), 11510 Puerto Real, Cadiz, Spain; ludisbel.leon@uca.es (L.L.-M.);

**Keywords:** polylactic acid, poly(3-hydroxybutyrate), poly(butylene succinate), polymers blended, supercritical solvent impregnation, *Mangifera indica* L. leaves

## Abstract

The present study evaluates the optical and colorimetric properties of Polylactic acid (PLA)-based films blended with Poly(3-hydroxybutyrate) (PHB) and Poly(butylene succinate) (PBS) and impregnated with mango leaf extract (MLE) using supercritical solvent impregnation (SSI) under different operating conditions (pressure: 10–30 MPa; temperature: 35–55 °C). Additionally, the relationship between impregnation load (IL) and color properties, as well as the release kinetics of the impregnated compounds, was investigated. The incorporation of PHB and PBS into the PLA matrix prior to impregnation led to a slight increase in the b* parameter (from 1.64 to 2.61), indicating a tendency toward yellowish tones. After SSI, all films exhibited noticeable color changes, with a shift toward yellowish-green hues and a decrease in lightness, regardless of processing conditions. Statistical analysis confirmed that polymer composition and its interaction with pressure and temperature significantly affected color properties (*p*-value < 0.001). The addition of PHB and PBS, as well as MLE impregnation, enhanced UV-barrier properties, while also modifying film transparency and opacity. In particular, PLA-PBS films showed higher opacity (more than 20 times) and lower transparency compared to neat PLA. These films also exhibited the highest IL values (2.41–4.75 mg MLE/100 mg polymer). Multivariate regression analysis demonstrated a strong correlation between CIELAB parameters (L*, a*, and b*) and IL (R^2^ > 85%, *p*-value < 0.001). Release studies in a food simulant showed partial release profiles, well described by Peleg’s model (R^2^ > 0.90). Furthermore, Korsmeyer–Peppas model fitting yielded diffusion exponents (n < 0.5), indicating quasi-Fickian diffusion mechanisms governing the release process.

## 1. Introduction

Global plastic production exceeded 400 million tonnes in 2025 [1], thereby contributing to increased environmental pollution. Biodegradable bioplastics have emerged as a sustainable alternative and are gaining increasing relevance across various sectors [2]. Current estimates indicate that global bioplastic production capacity will reach approximately 4.69 million tonnes by 2030, with an estimated growth of around 11% in the packaging segment [3,4]. Among biodegradable polymers derived from renewable resources, poly(lactic acid) (PLA), the family of poly(hydroxyalkanoates) (PHAs) and poly(butylene succinate) (PBS) are particularly noteworthy [2,5,6].

PLA is a biodegradable polyester [7] and currently the most widely used bio-based polymer in the food packaging industry [2,6]. It is derived from renewable resources such as sugar beet, sugarcane, corn and potatoes [7]. PLA exhibits favorable properties including good mechanical strength, biocompatibility, biodegradability and particularly high compostability [2,8]. Moreover, it has been classified as Generally Recognized as Safe (GRAS) by the U.S. Food and Drug Administration (FDA). PLA is a non-volatile, odorless and environmentally friendly polymer [2,7]. Additional advantages include high transparency, relatively high CO_2_ permeability compared to conventional petroleum-based polymers and good thermal processability, making it especially suitable for food packaging applications [7]. However, its broader application in this field is restricted by certain limitations, such as low flexibility, a slow crystallization rate and suboptimal barrier properties. Consequently, blending PLA with other polymers is a common strategy to enhance its performance [2].

In this context, PLA/poly(3-hydroxybutyrate) (PHB) blends have attracted significant attention. This combination allows the development of materials with improved performance while preserving their biodegradability [9]. PHB is an aliphatic polyester from the PHA family, produced by microorganisms as a carbon and energy storage material under nutrient-limited conditions [10,11]. PHB is characterized by high crystallinity and brittleness [11], as well as limited elasticity [10]. However, when combined with PLA, it can compensate for PLA’s low crystallinity and enhance barrier properties, which are crucial for food packaging applications [12].

Another approach to improve the processability and ductility of PLA involves blending it with PBS [13]. PBS is a biodegradable thermoplastic polymer that also belongs to the family of aliphatic polyesters [14] and can be produced from renewable resources such as sugarcane, cassava and corn [5]. This polymer is notable for exhibiting properties comparable to those of conventional petroleum-based plastics, along with versatile processability and good thermal stability [14]. In addition, the literature indicates that PLA-PBS blends offer numerous benefits, including the ability to enhance the ductility of PLA [5,13]. This is due to the action of PBS as a plasticizing agent, which improves tensile properties such as elongation at break and facilitates PLA crystallization [5].

Although the evaluation of the enhanced properties achieved through polymer blending (such as physical, mechanical, thermal, morphological and crystallinity characteristics) has been extensively reported, other relevant properties, including transparency for film applications, have received comparatively less attention [13]. For instance, PLA exhibits relatively low resistance to UV radiation [15]. In contrast, PHB and PBS generally show lower transparency [16,17], which may contribute to improved UV-blocking performance when incorporated into PLA matrices.

On the other hand, these polymer blends have been explored for the development of active films [6,18,19]. Active packaging systems are designed to release specific substances such as antioxidants, antimicrobial agents, carbon dioxide, flavor compounds, ethylene, or ethanol from the packaging material into the food or its surrounding environment [20], with the aim of extending shelf life and preserving or improving food quality [21]. In this context, phenolic compounds are particularly attractive as active agents due to their well-established antioxidant and antimicrobial properties [22,23]. Plant extracts constitute an important source of such bioactive compounds, especially phenolic constituents [20]. For instance, mango leaf extract is recognized as a rich source of polyphenols with significant antioxidant and antimicrobial activity [24,25]. Among its major bioactive constituents are mangiferin, gallic acid, quercetin derivatives, and iriflophenone derivatives, compounds that have been associated with high antioxidant activity [24]. In addition, several studies have reported the antimicrobial potential of mango leaf extract against different food-related microorganisms, highlighting its potential application as a natural active agent in food packaging systems [26].

Additionally, the incorporation of natural extracts into polymer matrices for the development of active films can significantly modify both optical and chromatics properties. For instance, Pan Fangya et al. reported improved UV-shielding properties in PLA films containing *Phoebe zhennan* extracts [15]. In a similar study, Arrieta et al. observed changes in light transmission and colorimetric properties in ternary PLA–PHB–limonene blends intended for biodegradable food packaging applications [27]. Generally, modifications in the microstructure of the films, along with the intrinsic color of the incorporated compounds, can alter light–material interactions. These changes may lead to increased light scattering and/or selective absorption of specific wavelengths, thereby affecting the overall optical properties of the films [28].

Polymeric films for active food packaging can be produced and functionalized through different fabrication and impregnation techniques, including solvent casting, extrusion, melt blending, compression molding, electrospinning [22,29] and supercritical fluid impregnation [30,31], among others. However, it is important to note that some film-forming techniques, such as extrusion, thermoforming and electrospinning, may require elevated processing temperatures [22] that can affect the stability of thermosensitive antioxidant compounds. Among these approaches, solvent casting is widely employed at laboratory scale due to its simplicity, low processing temperatures compared with melt-processing techniques, suitability for incorporating thermolabile bioactive compounds and relatively low equipment cost. This method involves dissolving the polymer mixture in a solvent, followed by mechanical mixing and solvent evaporation [29]. Nevertheless, the environmental impact associated with solvent emissions and hazardous waste generation limits its scalability and sustainability.

Alternatively, supercritical fluid impregnation has emerged as a promising strategy for the incorporation of active compounds into previously formed polymeric matrices [30,32]. This technique enables the incorporation of active compounds into polymeric matrices using a fluid in its supercritical state, most commonly carbon dioxide (scCO_2_) [31]. This solvent is widely used due to its chemical inertness, non-flammability, low cost and absence of taste and odor, as well as its relatively mild critical conditions [33]. Supercritical solvent impregnation (SSI) offers several advantages, including the prevention of thermal degradation of thermolabile compounds, homogeneous distribution of active agents within the polymer matrix, tunable loading by adjusting operating parameters, reduced processing times, minimal waste generation and the elimination of post-drying steps [31,32]. Several studies have highlighted the potential of SSI for the incorporation of mango leaf extract into different polymeric matrices. In summary, Cejudo-Bastante et al. investigated the impregnation of mango leaf extract into polymers such as acrylonitrile butadiene styrene (ABS), polyethylene terephthalate glycol (PETG), thermoplastic polyurethane (TPU), polycarbonate (PC), and polycaprolactone (PCL), demonstrating their potential for food and biomedical applications [34]. Likewise, comparative studies between SSI and solvent casting applied to bio-based nanocellulose films revealed improved antioxidant and antimicrobial performance for films produced through SSI [26]. In addition, León-Marcos et al. (2025) reported successful impregnation of mango leaf extract into PLA-PHB matrices, obtaining films with promising properties for active packaging applications [35].

On the other hand, previous studies have demonstrated correlations between impregnation loading and colorimetric properties in polymeric systems impregnated with natural extracts via SSI. Cejudo-Bastante et al. established a correlation between supercritical impregnation loading of olive leaf extract and color coordinates in polyethylene terephthalate (PET)/polypropylene (PP) films, highlighting the potential of color changes as a rapid and non-destructive indicator of active compound incorporation. However, this relationship has not yet been explored in PLA-based blended systems [36].

In this context, the present work investigates PLA-based films blended with PHB and PBS and impregnated with mango leaf extract via supercritical solvent impregnation under different processing conditions. The study evaluates the influence of polymer blending and SSI parameters on the optical and colorimetric properties of the materials, as well as on the impregnation loading achieved in the films. Furthermore, this work provides a detailed analysis of the correlation between CIELAB color coordinates and impregnation loading in PLA-based blended systems impregnated with mango leaf extract (MLE). These findings highlight the potential of colorimetric parameters as rapid and non-destructive indicators of impregnation in biodegradable active packaging materials.

## 2. Materials and Methods

### 2.1. Raw Materials

*Mangifera indica* L. leaves (Ken variety) used in the study were supplied by the Institute of Subtropical and Mediterranean Horticulture “La Mayora” (Malaga, Spain). The PLA polymer (heat deflection temperature at 1.8 MPa: 55 °C; melting point ≈ 150–170 °C; estimated molecular weight ≈ 80,000–150,000 g/mol; density: 1.24 g/cm^3^) was supplied in pellet form (nominal granule size: 3.0–5.0 mm) from Goodfellow (Hamburg, Germany). PBS (estimated molecular weight ≈ 80,000–120,000 g/mol; melting point: 114 °C; density: 1.27 g/cm^3^) was purchased in pellet form (nominal granule thickness: 2.60 ± 0.32 mm and nominal granule size: 3.31 ± 0.17 × 4.06 ± 0.18 mm) from Ningbo Neon Lion Technology Co., Ltd. (Ningbo, China). PHB (melting point = 172 °C; estimated molecular weight ≈ 750,000–1500,000 g/mol) was supplied in powder form from Sigma-Aldrich (Steinheim, Germany). Partially denatured ethanol (96% *v*/*v*) and absolute ethanol (99.9%) were supplied by PanReac AppliChem (Barcelona, Spain). Dichloromethane (≥99.8%) and carbon dioxide (99.99% purity) were provided by VWR BDH Prolabo (Catalonia, Spain) and Carburos Metálicos (Barcelona, Spain), respectively.

### 2.2. Film Preparation

All films were developed using the solvent casting technique. The procedure followed for each of them is described below:

PLA films: PLA was dissolved in dichloromethane (DCM) under constant stirring at 1000 rpm in glass bottles. A magnetic stirrer equipped with heating (J.P. Selecta, Barcelona, Spain) was used to facilitate the dissolution process, although heating was not required in this case. After complete dissolution of the polymer in DCM, approximately 6–7 mL of the polymer solution was poured onto a glass plate (inner diameter = 47.85 mm) under an extraction hood. The extraction hood was immediately turned off and the films were left to dry for 24 h at room temperature.

PLA-PHB films: The procedure followed was previously described by León-Marcos et al. [35]. PLA and PHB were dissolved separately in DCM under continuous stirring at 1000 rpm. To ensure the dissolution of PHB, it was necessary to heat it to a temperature of 65 °C. After complete dissolution, both polymer solutions were combined and further stirred under heating for 60 min to guarantee homogeneity. PLA-PHB blend films were prepared at a ratio of 75:25 (*w*/*w*). Subsequently, approximately 6–7 mL of the final polymer solution was cast onto a glass plate (inner diameter = 47.85 mm) under an extraction hood. The extraction hood was immediately turned off and the films were left to dry for 24 h at room temperature.

PLA-PBS films: These films were prepared following the same procedure described for the PLA-PHB films. In this case, heating the PBS solution in DCM to 65 °C was also required to ensure complete dissolution. Similarly, PLA-PBS blend films were prepared at a ratio of 75:25 (*w*/*w*).

For comparison purposes, films composed of pure PHB and pure PBS were also developed. All obtained films were finally stored at room temperature until further characterization and impregnation experiments.

### 2.3. Supercritical Extraction of Mango Leaf Extract

The mango leaf extract (MLE) was obtained following an enhanced extraction procedure previously reported by Fernández-Ponce et al. (2021) [37]. This method employed a solvent mixture composed of carbon dioxide and ethanol (CO_2_:EtOH 50% *v*/*v*). The extraction was performed using 200 g of dried mango leaves, previously ground, placed in a cartridge and loaded into a 1000 mL high-pressure extraction vessel (SF1000, Thar Technologies, Pittsburgh, PA, USA). The process was conducted at 20 MPa, 80 °C in batch mode for 24 h. The resulting ethanolic extract presented a concentration of 82 ± 2 mg/mL and an extraction yield of 20 ± 0.3%. The total phenolic content was 124.73 ± 13.44 μg GAE/mg dried material, while the antioxidant activity index (AAI) was 2.06 μg DPPH/μg extract. The mango leaf extract was subsequently stored at 15 °C until further use.

### 2.4. Impregnation of Bioactive Films

**Figure 1:**
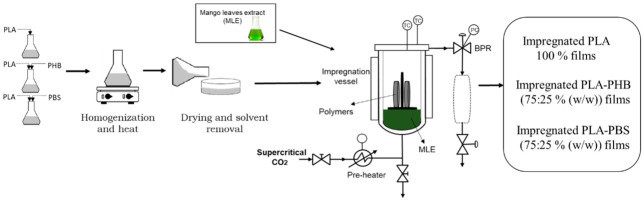
Schematic representation of the film preparation and Supercritical solvent impregnation (SSI) procedures for Poly(lactic acid) (PLA), PLA-Poly(3-hydroxybutyrate) (PHB) and PLA-Poly(butylene succinate) (PBS) blends.

Impregnation assays were performed in batch mode, following the methodology reported by León-Marcos et al. using a RESS250 laboratory-scale unit (Thar Technologies, Pittsburgh, PA, USA). This system includes a 250 mL high-pressure vessel with a thermostatic jacket and a CO_2_ storage tank, condenser, high-pressure pump, heat exchanger and back-pressure regulator (BPR) with a micrometric valve [38]. Two film samples with an identical polymer composition were placed inside the impregnation vessel using a stainless steel holder. Additionally, 6 mL of MLE was introduced into the vessel. The films were carefully arranged to avoid contact with each other, ensuring uniform exposure of all surfaces to the supercritical CO_2_. We also verified that the polymer samples were not in direct contact with the liquid extract. The impregnation setup is schematically illustrated in Figure 1.

A series of experiments were conducted to evaluate the effect of pressure (10, 20 and 30 MPa) and temperature (35 and 55 °C) on the chromatic and optical behavior of different PLA-based film formulations (PLA, PLA-PHB and PLA-PBS). The depressurization rate was kept constant at 0.1 MPa/min.

### 2.5. Thickness of the Films Measure

The film thickness was measured using a handheld digital micrometer (MDC-25PX, Mitutoyo Corporation, Tokyo, Japan) with an accuracy of 1 μm. To assess potential variations across the film surface, three thickness measurements were obtained for each film.

### 2.6. Impregnation Loads (IL) of MLE in Polymers

The impregnation load was quantified by dissolving a known amount of the impregnated material in dichloromethane (DCM) at a concentration of 1.1 mg of polymer/mL of solvent until complete dissolution was achieved. The resulting solutions were then subjected to ultrasonic treatment for 15 min to ensure full dissolution of the polymers and the impregnated compounds. Subsequently, the absorbance of each sample was measured at 314 nm using a UV–Vis spectrophotometer (Hach Lange DR 500, Düsseldorf, Germany). The impregnation load was determined from calibration curves previously established for both the extract and the polymer matrices:

Ethanolic mango leaf extracts (0.005–0.5 mg/mL) calibration lines in DCM:

Abs (314 nm) = 10.24·(MLE(mg/mL)) + 0.03         R^2^ = 0.99 (1)

PLA (0.46–1.54 mg/mL) calibration lines in DCM:

Abs(314 nm) = 0.004·(PLA (mg/mL)) + 0.0008        R^2^ = 0.99 (2)

PLA-PHB (0.28–1.10 mg/mL) calibration lines in DCM:

Abs(314 nm) = 0.26·(PLA-PHB(mg/mL)) + 0.03        R^2^ = 0.99 (3)

PLA-PBS (0.46–1.60 mg/mL) calibration lines in DCM:

Abs(314 nm) = 0.003·(PLA-PBS(mg/mL)) + 0.002       R^2^ = 0.99 (4)

The impregnation load is expressed as mg MLE/100 mg polymer.

### 2.7. Chromatic and Optical Characterization

In order to evaluate the optical and colorimetric effects associated with the incorporation of PBS and PHB into PLA matrices, as well as their influence on the impregnation process, several optical parameters were analyzed. These included color properties within the CIELAB color space, chroma (C*ab), hue angle (h*ab), whiteness index (WI) and yellowness index (YI), as well as transmittance, opacity and transparency.

The CIELAB color coordinates (L*, a* and b*) were determined, where L* represents lightness (ranging from 0 for black to 100 for white), a* denotes the green–red axis (negative values indicating green and positive values indicating red) and b* corresponds to the blue–yellow axis (negative values indicating blue and positive values indicating yellow) [39]. Measurements were performed in triplicate at randomly selected positions on each film using a portable spectrophotometer (CM-2600d, Konica Minolta, Tokyo, Japan).

The total color difference (Δ*E*) between impregnated and non-impregnated samples was calculated according to Equation (5):

(5) $$∆E=\sqrt{{\left({∆a}^{*}\right)}^{2}+{\left({∆b}^{*}\right)}^{2}+{\left({∆L}^{*}\right)}^{2}}$$

where ΔL*, Δa* and Δb* represent the differences in the corresponding color parameters between impregnated and non-impregnated films.

In addition, C*ab, h*ab, WI and YI were calculated using the following equations [40,41]:

(6) $${C^{*}}_{ab}=\sqrt{{a^{*}}^{2}+{b^{*}}^{2}}$$

(7) $${h^{*}}_{ab}=arctg\left(\frac{a^{*}}{b^{*}}\right)$$

(8) $$WI=100-\sqrt{{\left(100-L^{*}\right)}^{2}+{a^{*}}^{2}+{b^{*}}^{2}}$$

(9) $$YI=\frac{142.86}{L^{*}}$$

The optical transmittance of the films was measured using a UV–Vis spectrophotometer (UV-1800, Shimadzu, Kyoto, Japan). Film samples (1 × 4 cm) were placed in a quartz cuvette and analyzed over the wavelength range of 200–800 nm, with a step size of 5 nm. The transparency and opacity of the films were calculated according to Equations (10) and (11) [40], respectively:

(10) $$Transparency\;({mm}^{-1})=\frac{\log{\%T}_{600}}{x}$$

(11) $$Opacity\;({mm}^{-1})=\frac{2-\log{\%T}_{600}}{x}$$

where %T_600_ is the percentage transmittance at 600 nm and x is the film thickness.

### 2.8. Release of Impregnated Active Compounds

In order to evaluate the differences in the release behavior of the impregnated compounds from the developed films (PLA, PLA-PHB and PLA-PBS), release experiments were conducted using samples previously impregnated at 20 and 30 MPa and 35 °C. These conditions were selected based on the impregnation loading results in order to assess the release of active compounds into a food simulant.

The food simulant D1 (ethanol:water 50% (*v*/*v*)) was employed, as it is recommended for alcoholic foods (alcohol content higher than 20%) and oil-in-water emulsions [42]. Impregnated film samples were immersed in the simulant at an approximate concentration of 2 mg of film per mL of simulant and placed in an incubator (New Brunswick Scientific, Edison, NJ, USA, Excella E24R model) at 40 °C for 32 days.

The absorbance of the release medium was measured at 314 nm at predetermined time intervals using a UV–Vis spectrophotometer (UVmini-1240, Shimadzu, Sydney, Australia). Prior to the release experiments, a calibration curve of mango leaf extract in ethanol:water 50% (*v*/*v*) was established within the concentration range of 0.0001–0.10 mg/mL. The corresponding calibration equation was:

(12) $$Abs\;(314\;nm)=8.25\cdot (MLE(mg/mL))+0.01\;\;\;\;\;R^{2}=0.99$$

The amount of released compounds was expressed as the final concentration relative to 100 mg of polymer.

To describe and predict the release kinetics of the active compounds, six empirical models were fitted to the experimental release data: zero-order, first-order, Higuchi, Korsmeyer–Peppas (Power Law), Peleg and Avrami models (Equations (13)–(18), respectively) [43,44]. In these models, *M_t_*, *M_o_* and *M_∞_* are the cumulative amounts of active compound released at time *t*, the maximum amount (the total impregnation load) and at infinite time, respectively; *k* is the kinetic constant; 1/*k*_2_ is the ratio of active compound released at equilibrium (M_∞_/M_o_); and 1/*k*_1_ is the initial release rate and *n* the released or diffusional exponent [45,46]. The mass of active released at equilibrium (M_∞_) was determined from Peleg’s model [47,48]. All model fittings were performed using OriginPro 2024 SR1 software and the Excel Solver tool (Microsoft Excel 2016).

(13) $$Zero\text{-}order\;\;\;\;\;\;\;\;\;\;\;\;\;\;\;\;\;\;\;\;\;\;\;\;\;\;\;\;\;\;\;\;\;\;\;\;\;\;\;\;\;\;\;\;\;\;\;\;\;\;\;\;\;\;\;\;\;\;\;\;\;\;\;\;\;\;\;\frac{M_{t}}{M_{\infty }}=kt$$

(14) $$First\text{-}order\;\;\;\;\;\;\;\;\;\;\;\;\;\;\;\;\;\;\;\;\;\;\;\;\;\;\;\;\;\;\;\;\;\;\;\;\;\;\;\;\;\;\;\;\;\;\;\;\;\;\;\;\;\;\;\;\;\;\;\frac{M_{t}}{M_{\infty }}=1-e^{kt}$$

(15) $$Higuchi\;\;\;\;\;\;\;\;\;\;\;\;\;\;\;\;\;\;\;\;\;\;\;\;\;\;\;\;\;\;\;\;\;\;\;\;\;\;\;\;\;\;\;\;\;\;\;\;\;\;\;\;\;\;\;\;\;\;\;\;\;\;\;\;\;\;\;\frac{M_{t}}{M_{\infty }}=kt^{1/2}$$

(16) $$PowerLaw/Korsmeyer–Peppas\;\;\;\;\;\;\;\;\;\;\;\;\;\;\;\;\;\;\;\;\;\;\;\;\;\;\;\;\frac{M_{t}}{M_{\infty }}=kt^{n}$$

(17) $$Peleg\;\;\;\;\;\;\;\;\;\;\;\;\;\;\;\;\;\;\;\;\;\;\;\;\;\;\;\;\;\;\;\;\;\;\;\;\;\;\;\;\;\;\;\;\;\;\;\;\;\;\;\;\;\;\;\;\;\;\;\;\;\frac{1}{{M_{t}/M}_{0}}=k_{1}-k_{2}t$$

(18) $$Avrami\;\;\;\;\;\;\;\;\;\;\;\;\;\;\;\;\;\;\;\;\;\;\;\;\;\;\;\;\;\;\;\;\;\;\;\;\;\;\;\;\;\;\;\;\;\;\;\;\;\;\;\;\;\;\;\;\;\;\;\;\frac{M_{t}}{M_{\infty }}=1-e^{\left(-kt^{n}\right)}$$

The application of these models was based on several assumptions: (i) the volume of the release medium is finite and remains constant throughout the experiment [44]; (ii) interactions between the food simulant and the polymer matrix are negligible; (iii) no structural changes occur in the films during the release process; (iv) the initial concentration of active compounds in the simulant is zero; (v) the active compounds are homogeneously distributed within the films at the beginning of the experiment [49]; (vi) no degradation of the active compounds occurs during the initial and exponential release stages.

### 2.9. Statistical Analysis

All results are expressed as the mean of at least three replicates, together with their corresponding confidence intervals. Statistically significant differences among samples are indicated by different letters in tables and figures. Statistical comparisons were performed using Fisher’s least significant difference (LSD) test at a 95% confidence level.

A multilevel factorial experimental design was applied to evaluate the effect of incorporating PHB and PBS into PLA-based formulations, as well as the influence of pressure and temperature during the impregnation process on impregnation loading, opacity, transparency and color parameters. Interaction effects were considered up to the second order.

In addition, multiple linear regression analysis was conducted to establish the relationship between the color parameters of each polymer formulation and their corresponding impregnation loading.

All statistical analyses were carried out at a 95% confidence level using Statgraphics 19-X64 software (version 19.6.02).

## 3. Results

In the present study, PLA, PLA-PHB and PLA-PBS blends were developed and subsequently impregnated with MLE using SSI technology. The main objective was to evaluate the optical and colorimetric properties of the films, focusing on the influence of PHB and PBS incorporation into PLA, as well as the effect of different pressure and temperature conditions during the impregnation process.

A PLA-PHB ratio of 75:25% (*w*/*w*) was selected based on previous studies reporting significantly improved tensile properties compared to neat PLA. This enhancement has been attributed to the presence of finely dispersed PHB crystals, which act as reinforcing fillers and nucleating agents within the PLA matrix [50]. While the evaluation of mechanical and crystallinity properties is not the primary focus of this work, the use of polymer blends with improved performance is considered advantageous. For the PLA-PBS blends, the same polymer ratio (75:25% *w*/*w*) was employed to allow a direct comparison with the PLA-PHB system, although previous studies have also reported PLA-PBS blends with similar compositions [51].

**Figure 2:**
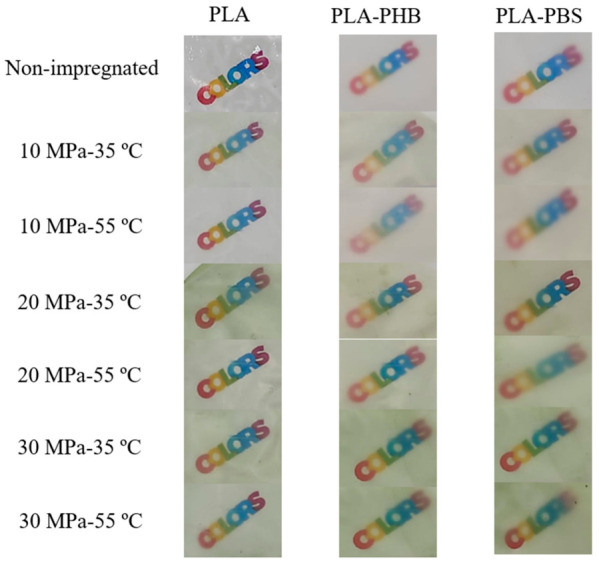
Photographs of the PLA, PLA-PHB and PLA-PBS films before and after mango leaf extract (MLE) impregnation via SSI. A white background paper with the word “colors” written with different colors was used.

Figure 2 presents photographs of both impregnated and non-impregnated PLA, PLA-PHB and PLA-PBS films. Prior to the impregnation process, neat PLA films exhibited high transparency. However, the incorporation of PHB and PBS resulted in visibly more opaque films. After impregnation with MLE via SSI, homogeneous films were successfully obtained with noticeable changes in color and tonalities observed across all formulations.

The thickness of the films (Table 1) prior to impregnation was approximately 0.06 ± 0.001 mm, regardless of polymer composition. After impregnation, the thickness of the PLA and PLA-PHB films ranged between 0.07 and 0.10 mm, whereas PLA-PBS films showed values between 0.06 and 0.09 mm. Despite these variations, no statistically significant differences in thickness were identified among the samples.

It is worth noting that PLA-PBS films impregnated at 10 MPa and 55 °C exhibited slight permanent swelling in certain regions of the film, which explains the higher thickness values observed for this formulation.

### 3.1. Impact on Chromatic Properties on PLA, PLA-PHB and PLA-PBS Films Impregnated via SSI

The incorporation of the biopolymers PHB and PBS into the PLA matrix resulted in a slight increase in the b* parameter, indicating a tendency toward yellowish tones in the films (Table 2). Arrieta et al. also reported color changes in PLA-PHB systems; however, in their study, the initial PHB exhibited a pronounced yellow coloration, with b* values exceeding 22 [27]. Compared with these results, the PHB used in the present study showed significantly lower a*, b* and L* values (a* = −1.48 ± 0.10, b* = 2.83 ± 0.27 and L* = 90.14 ± 0.89) prior to blending. Similarly, Siracusa et al., in a study on PLA-PHB blends prepared by solvent casting, observed a slight shift toward yellowish coloration compared to neat PLA [52]. In agreement with these findings, Coltelli et al. reported a slight increase in b* values and a decrease in a* and L* in PLA-based films upon the addition of poly(butylene succinate-co-adipate) (PBSA), a copolymer of PBS with an adipic acid [53].

The impregnation process with MLE induced significant color changes in all films (Figure 2 and Table 2), characterized by a shift toward yellowish-green tones and a decrease in the lightness parameter (L*), regardless of the operating conditions. The total color difference (Δ*E*) between non-impregnated and impregnated samples was greater than 4. According to the literature, Δ*E* values between 3.5 and 5 indicate clearly noticeable differences, while values above 5 correspond to distinctly different colors [39,54]. Other studies also report that Δ*E* values greater than 3 CIELAB units are perceptible to the human eye [55]. According to the established criteria, the impregnation process altered the polymer’s color, resulting in a difference that is readily apparent to the observer, who perceives two distinct colors.

These results are consistent with those of previous studies which have demonstrated that incorporating natural extracts into polymer matrices can significantly affect the color of films [56,57,58]. For instance, Petaloti et al. reported a decrease in lightness (L*) and an increase in a* and b* values in PLA films containing coffee silverskin extracts [57]. Verano-Naranjo et al. conducted a study on the supercritical impregnation of PLA/poly(butylene adipate-co-terephthalate)/thermoplastic starch films with olive leaf extract. The impregnated films exhibited a strong presence of green and yellow tones, along with reduced L*, a* and b* values, which were associated with the impregnation loading [58].

The observed color changes can be attributed to the chemical composition of the mango leaf extract. Mango leaves contain significant amounts of chlorophyll, which gives them their green color [54], as well as a wide range of phenolic compounds [24,59,60,61] that can generate yellow to brown tones [54]. In line with these findings, Cejudo et al. also reported similar trends in the color properties of nanocellulose films impregnated with mango leaf extracts [26].

Furthermore, variations in impregnation conditions also influenced the color of the films. More intense colorations and a more pronounced decrease in L* were observed under operating conditions that resulted in higher impregnation loadings. This indicates a relationship between the amount of incorporated extract and the resulting optical properties.

**Figure 3:**
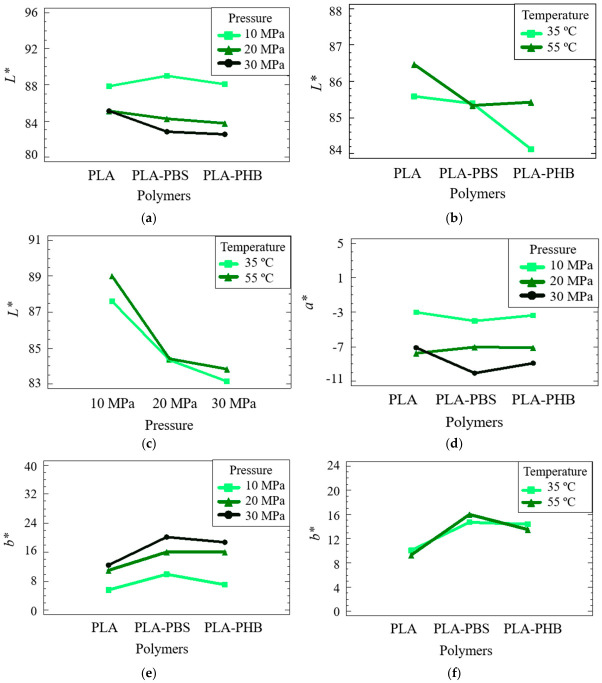
Interactions plot of the variation in pressures and polymer blend (**a**,**d**,**e**), temperature and polymer blend (**b**,**f**), pressures and temperatures (**c**) on the L* (**a**–**c**), a* (**d**) and b* (**e**,**f**) parameters.

The variation in polymer formulation and its interaction with pressure were found to be statistically significant in the changes observed in the color properties of the impregnated films (*p*-value *=* 0.00) (Figure 3a,d,e and Tables S1–S3 in the Supplementary Material). Furthermore, the interaction between polymer blend and temperature was also significant for the L* and b* parameters (*p*-value *=* 0.00 in both cases) (Figure 3b,f and Tables S1 and S3 in the Supplementary Material).

For the L* parameter (Figure 3a), it was observed that at 10 MPa, the presence of PHB and PBS led to a slight increase in lightness. However, at higher pressures, the incorporation of PHB and PBS into PLA resulted in films with lower L* values, suggesting reduced lightness. Regarding the a* parameter (Figure 3d), a similar trend was observed at 10 and 30 MPa, with a greater tendency toward green tones in films containing PBS and PHB (with a* values more negative) compared to neat PLA. In contrast, at 20 MPa, a slight shift toward more positive a* values was observed in PLA-PHB and PLA-PBS films, although the effect was less pronounced. For the b* parameter (Figure 3e), both PLA-PHB and PLA-PBS blends exhibited increased values regardless of the pressure applied, indicating a shift toward yellow coloration. This trend became more pronounced with increasing pressure. These results suggest that while the incorporation of PBS and PHB into PLA before the impregnation process may induce slight variations in the b* parameter, the contribution of the extract during the impregnation process plays a more significant role in the observed color changes in the impregnated films.

Conversely, blending PLA with PHB or PBS generally resulted in a decrease in lightness at both temperatures examined, compared with pure PLA (Figure 3b). At 55 °C, analogous L* values were obtained for PLA-PHB and PLA-PBS films; however, at a lower temperature (35 °C), PLA-PHB films exhibited a more pronounced decrease in lightness. Furthermore, an increase in temperature resulted in higher L* values in PLA and PLA-PHB films. In contrast, no significant change in lightness was detected in PLA-PBS films. Conversely, the interaction between temperature and polymer composition on the b* parameter (Figure 3f) after impregnation exhibited similar trends at both temperatures, with an increase in this parameter for PLA blended with PHB and PBS.

The significant interaction between pressure and polymer composition can be explained by the dependence of the supercritical impregnation process on both CO_2_ density and polymer structure. Increasing pressure enhances the density of supercritical CO_2_, improving solute solubility and CO_2_ sorption into the polymer matrix [25,62], which induces swelling and plasticization phenomena [23,31,33]. As a result, polymer chain mobility and extract diffusion are enhanced [31], leading to higher impregnation loadings. This increased extract content is reflected in more pronounced color changes, typically associated with a decrease in lightness (L*) and an increase in the b* parameter. In general, higher extract loadings or differences in the incorporated active compounds result in noticeable variations in color coordinates [56,63], as previously reported for biodegradable polymers containing chromophoric additives [26,63,64].

Furthermore, polymer composition plays a key role in this behavior. While PLA is predominantly amorphous [65], the incorporation of semicrystalline polymers such as PHB and PBS [66,67] modifies the crystallinity and morphology of the material [68,69]. These structural differences affect CO_2_ uptake, swelling behavior and diffusion properties, influencing the amount and distribution of the extract within the matrix and, consequently, the resulting color of the films.

**Figure 4:**
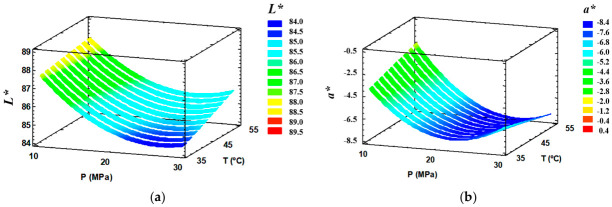

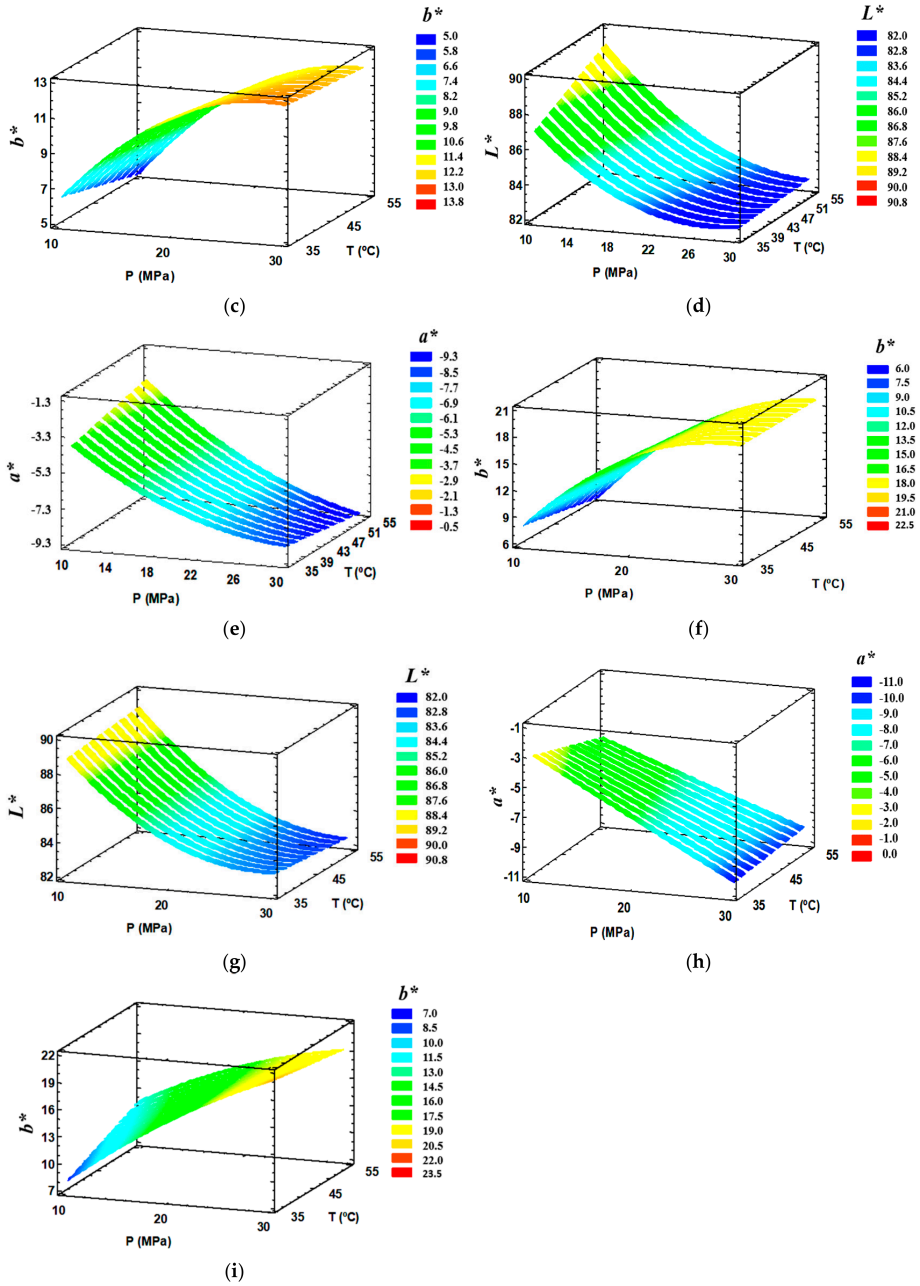
Estimated response surface for chromatic coordinates L* (**a**,**d**,**g**), a* (**b**,**e**,**h**) and b* (**c**,**f**,**i**) related to the impregnation temperature and pressure of the PLA (**a**,**b**,**c**), PLA-PHB (**d**,**e**,**f**) and PLA-PBS (**g**,**h**,**i**) polymers impregnated with MLE using SSI.

On the other hand, Figure 4 illustrates the combined effect of pressure and temperature on the color properties of each polymer system. Since different response surface profiles were observed for each formulation, it can be concluded that the impregnation process under selected conditions is influenced by structural differences between the polymers, resulting in variations in impregnation loading and, consequently, in the color properties of the resulting films.

A substantial increase in chroma values (Table 3) was observed in films prepared with the extracts; this increase was more than three times higher than in the non-impregnated films. Petaloti A-I. et al. also observed this phenomenon when they impregnated PLA films with coffee (CS) extracts [57]. An increasing trend in chroma values was observed with increasing impregnation pressure for all polymer formulations. However, films containing PBS and PHB exhibited higher chroma values at elevated pressures compared to impregnated neat PLA films. These results are consistent with the higher b* values and darker tones observed (Table 2), indicating an increase in color saturation at higher operating pressures.

Additionally, the hue angle (Table 3) was also significantly affected by the incorporation of the extract into the polymer matrix, demonstrating a decline in comparison to non-impregnated films. Similarly, the whiteness index significantly decreased after impregnation, indicating a general darkening of the films. Comparable trends have been reported in the literature for polymeric systems incorporating natural extracts [40,57].

Regarding the yellowness index (Table 3), an increase was observed in all impregnated films compared to non-impregnated films. A similar trend to that observed for chroma was detected, with YI values increasing as impregnation pressure increased. Although YI is commonly used to assess color changes associated with polymer degradation [70], in this case its increase is mainly attributed to the incorporation of the extract.

As previously discussed, MLE contains a wide variety of phenolic compounds [24,59,60,61], which can contribute to color variations, generating shades ranging from yellow to brown [54]. Among the main bioactive compounds present in MLE are quercetin and mangiferin [24,59,60,61], which have also been identified in supercritical impregnation processes of polyester-based films [35,71]. Previous studies have demonstrated that the incorporation of such compounds into polymer matrices can lead to yellowish colorations. For instance, Łopusiewicz et al. reported yellowish tones in PBS films impregnated with quercetin [72]. Additionally, Parhi et al. described the extraction and purification of mangiferin from *Mangifera indica* L. leaves, obtaining a light-yellow precipitate [73], which may further explain the contribution of this compound to the coloration of the films.

### 3.2. Optical Properties of PLA, PLA-PHB and PLA-PBS Impregnated Films via SSI

One of the key requirements in food packaging is protection against ultraviolet (UV) radiation, particularly for products susceptible to oxidative degradation [74]. Therefore, the UV-blocking ability of films is a critical parameter in the development of packaging materials for UV-sensitive foods [15].

**Figure 5:**
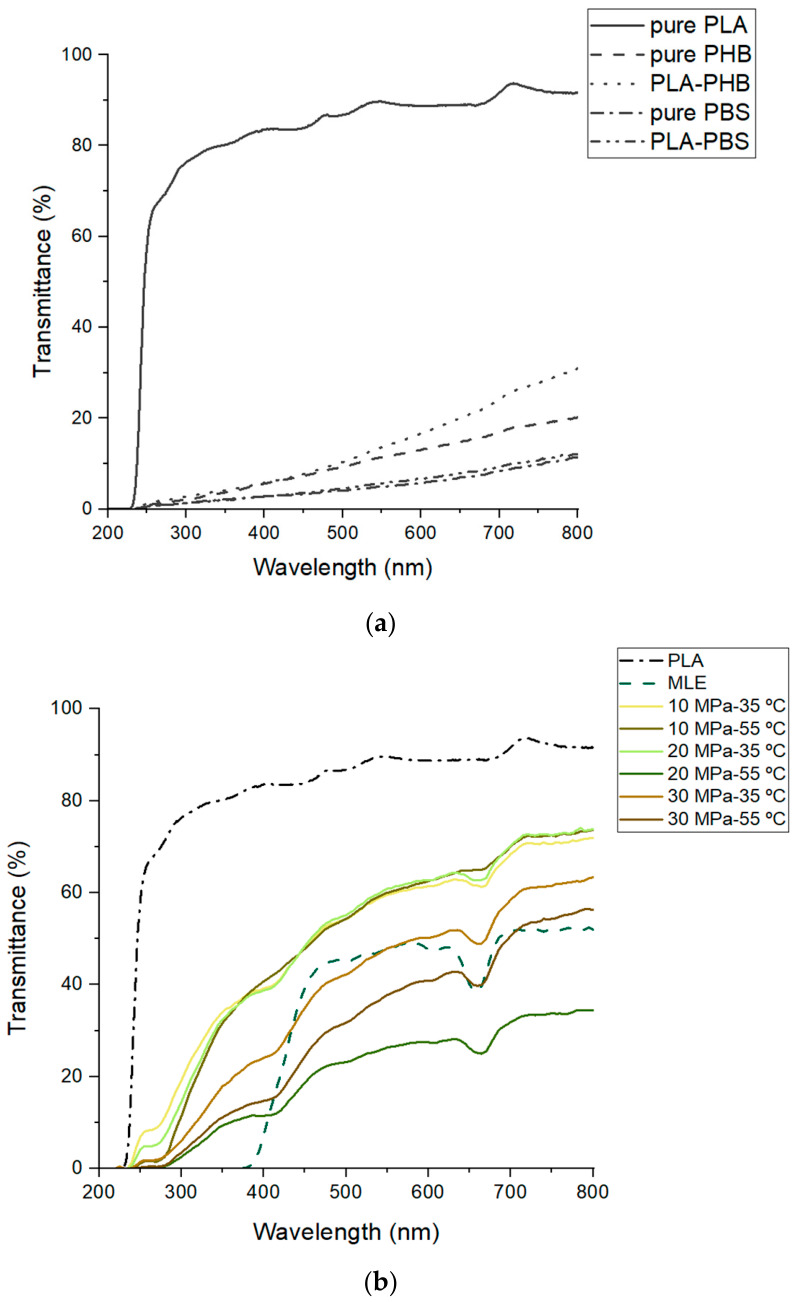

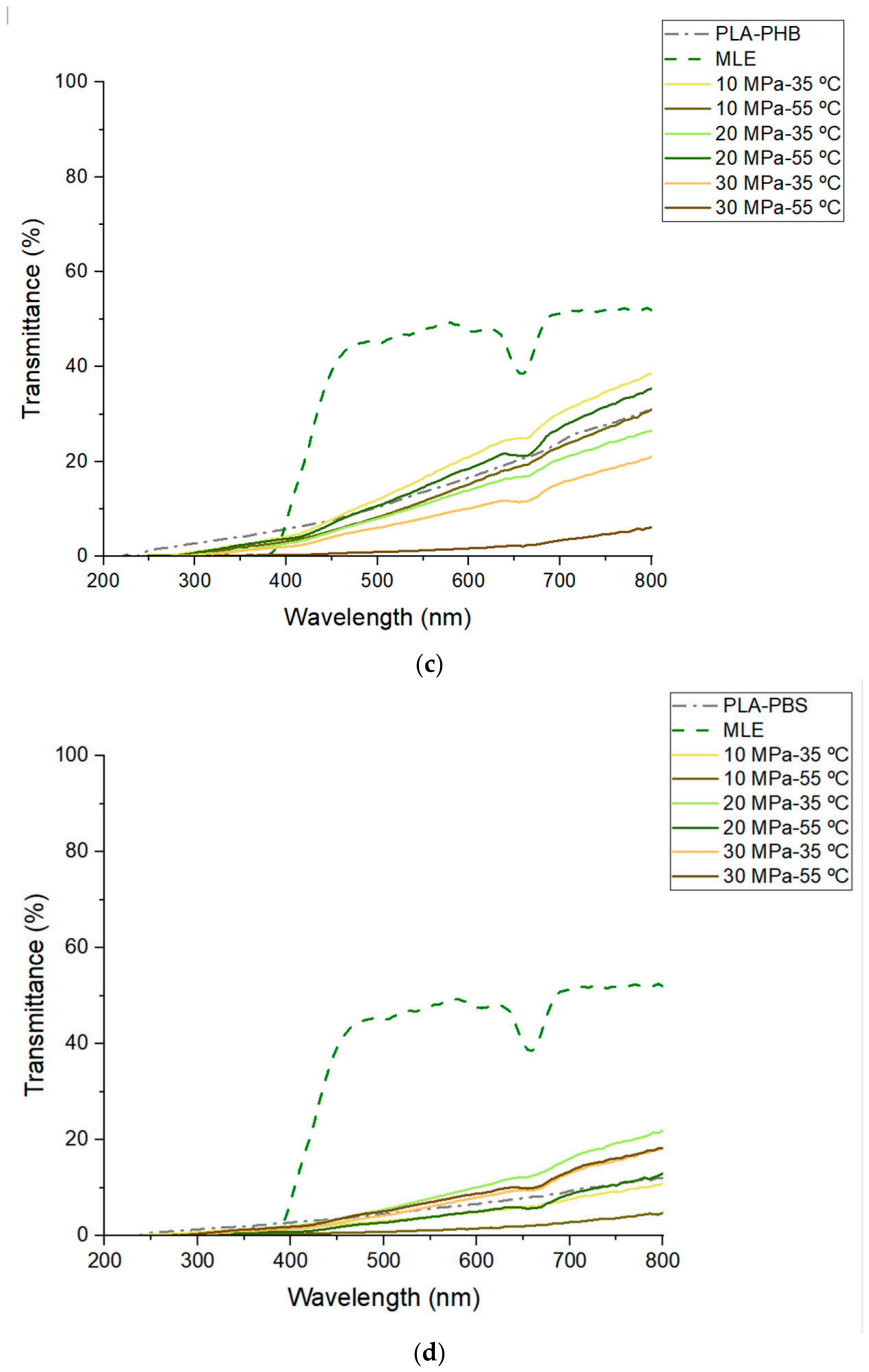
UV–Vis transmittance spectra of pure PLA, PHB, PBS and their blends (PLA–PHB and PLA–PBS) (**a**) and films impregnated with MLE: PLA (**b**), PLA–PHB (**c**) and PLA–PBS (**d**).

The light barrier properties of the films were evaluated by UV–Vis spectroscopy in the 200–800 nm range, considering two main regions: from 200 to 400 nm (UV spectrum) and from 400 to 800 nm (visible spectrum). As illustrated in Figure 5a, there is a decrease in transmittance percentage (%T) for PLA-based films blended with PBS and PHB compared to neat PLA. This can be associated with an enhancement in UV-light protection. It is recognized that the combination of PLA with other polymers, including PHB and PBS, provides enhanced UV protection compared to PLA films [6,17].

The PLA-PBS blend did not demonstrate notable variations in %T compared to the neat PBS. In contrast, PLA-PHB films demonstrated a different behavior, with a slight increase in %T above 550 nm compared to pure PHB. These results suggest that the observed differences may be related to the intrinsic properties of PHB and its interaction with PLA. One potential explanation for this phenomenon is the difference in refractive index between PHB (1.59 approximately) and PLA (1.45 approximately) [75], which may generate optical effects that increase transmittance at certain wavelengths. Additionally, PHB is a highly crystalline polymer with a natural tendency to form crystalline domains [52,76] that scatter light. When blended with PLA, the crystallinity of PHB may be reduced [12,27], leading to increased light transmission. Furthermore, the partial immiscibility between PLA and PHB [35,76] promotes phase separation; if the resulting domains are sufficiently small, light scattering may be reduced, thereby increasing transmittance.

The impregnation of the extract into neat PLA films (Figure 5b) improved UV-light protection regardless of the operating conditions. Specifically, conditions of 20 and 30 MPa at 55 °C and 30 MPa at 35 °C yielded the highest UV-blocking performance, with transmittance values ranging from 2 to 6% at 300 nm. However, these films also exhibited the lowest transmittance in the visible region. Similar behavior was reported by Miranda-Villa et al. (2022), who observed that the incorporation of R-carvone into PLA via supercritical CO_2_-assisted impregnation enhanced absorption in both UV and visible regions, indicating improved UV-barrier properties but low transparency in the visible range [77].

Similarly, the incorporation of the extract into PLA-PHB and PLA-PBS blends consistently enhanced UV protection (Figure 5c,d). At 300 nm, all impregnated samples exhibited transmittance values close to zero, indicating significantly improved UV-blocking capacity compared to the non-impregnated films. For the PLA-PHB blend (Figure 5c), the impregnation conditions resulted in differing levels of protection against UV light. Samples impregnated with 10 and 20 MPa exhibited an increase in transmittance above 300 nm, whereas those impregnated with 30 MPa maintained transmittance values close to 0 up to 400 nm, with %T of 0.3 to 3%, depending on the condition. Similarly, the transmittance values of PLA-PBS films (Figure 5d) were close to zero at 400 nm under all conditions tested, indicating enhanced UV protection after impregnation.

The low transmittance values exhibited by MLE-impregnated PLA-PHB and PLA-PBS films support their capacity to absorb UV radiation. Consequently, they can be designated as ultraviolet absorbers (secondary or preventive antioxidants), possessing the ability to prevent the photooxidation of photosensitive foods [26,78,79].

Nevertheless, the opacity and transparency are important and commonly measured characteristics of food packaging film [58,80], as they determine the visual appearance and light barrier capacity of films and are important in customer acceptance [58]. It is noteworthy that PLA is transparent and colorless, whereas PHB and PBS present a lower transparency [6,16,17,53]. The combination of these polymers with PLA has been demonstrated to enhance the visual characteristics of PHB and PBS [17,81]. Conversely, numerous studies have indicated that the incorporation of polyphenolic compounds or essential oils into the mixture can facilitate an improvement in the optical properties of the combined polymer [27,82]. However, the impregnation of natural extracts may result in an increase in opacity [58,80] which can affect the visual appearance.

**Figure 6:**
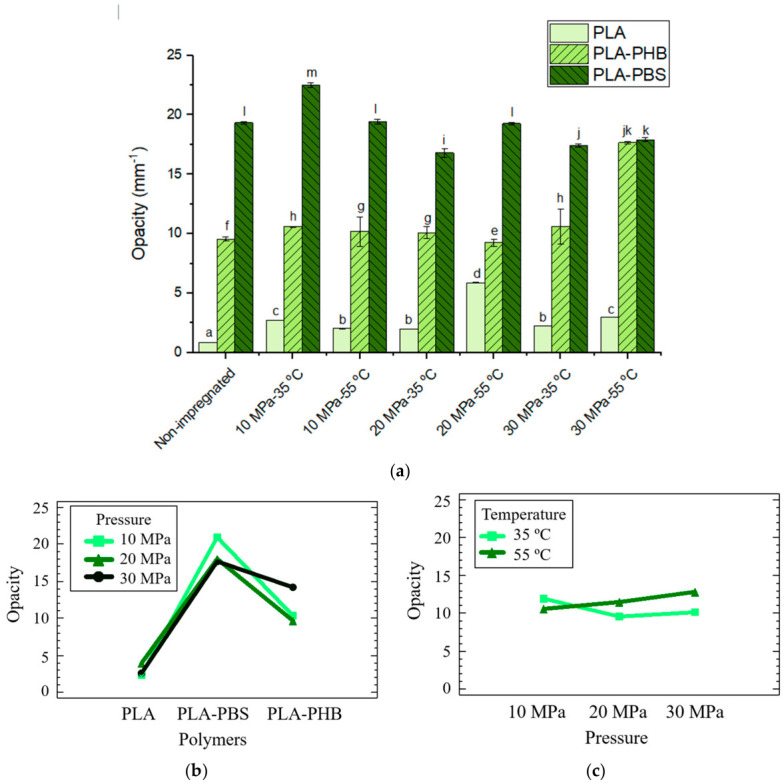
Opacity of PLA, PLA-PHB and PLA-PBS films before and after MLE impregnation (**a**) and interaction effects of pressure–polymer formulation (**b**) and pressure–temperature (**c**). The values are the mean of at least three replicates and the error bars correspond to the confidence interval. Different letters indicate significant differences.

**Figure 7:**
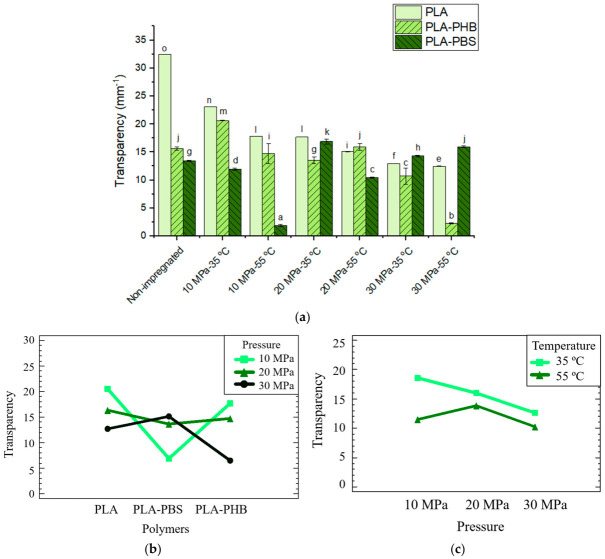
Transparency of PLA, PLA-PHB and PLA-PBS films before and after MLE impregnation (**a**) and interaction effects of pressure–polymer formulation (**b**) and pressure–temperature (**c**). The values are the mean of at least three replicates and the error bars correspond to the confidence interval. Different letters indicate significant differences.

As shown in Figure 6a and Figure 7a, both polymer blending and MLE impregnation markedly affected opacity and transparency of the films. The incorporation of PHB and PBS into the PLA matrix led to a significant increase in opacity (Figure 6a), with opacity values more than 10 and 20 times higher, compared to pure PLA. Consequently, the transparency of the PLA-PHB and PLA-PBS films decreased by 107% and 142%, respectively, compared to pure PLA (Figure 7a).

A similar trend was observed after the impregnation of PLA films with MLE, where an increase in opacity and a corresponding decrease in transparency were detected (Figure 6a and Figure 7a). Comparable behaviors have previously been reported for PLA films impregnated with natural extracts, and these effects have been associated with differences in the refractive indices of PLA and the impregnated active compounds, which modify light scattering and absorption within the films and consequently affect light transmittance [83].

The opacity and transparency values of PLA-PHB films (Figure 6a and Figure 7a) were not significantly affected by MLE impregnation at 10 and 20 MPa when compared to the non-impregnated films. However, under impregnation conditions of 30 MPa and 55 °C, an increase of more than two times in opacity was observed, along with a reduction in transparency of more than sixfold relative to the neat film. Although these conditions also resulted in high impregnation loadings, this behavior may additionally be attributed to polymer chain rearrangement and interactions between the impregnated compounds and the polymer matrix.

For PLA-PBS films (Figure 6a), opacity values did not show substantial differences among the different conditions tested. Nevertheless, a significant decrease in transparency was observed when the films were impregnated at 10 MPa and 55 °C (Figure 7a). This result may be closely related to the structural changes observed under these conditions, where localized permanent swelling occurred in certain regions of the film, affecting light transmission properties.

The interaction between polymer type and pressure, as well as between pressure and temperature, was found to be statistically significant in the variation in opacity (Figure 6b,c and Table S4 in the Supplementary Material) and transparency (Figure 7b,c and Table S5 in the Supplementary Material) of films impregnated with MLE (*p*-value = 0.00 in all cases). The incorporation of PBS into PLA, followed by impregnation, resulted in films with higher opacity and lower transparency across all pressures evaluated.

Variations in pressure and temperature during the supercritical impregnation process may induce rearrangement of polymer chains due to the plasticizing effect of CO_2_ and the associated swelling phenomena [31,33], leading to changes in optical behavior. Additionally, during the SSI process, crystalline regions may partially melt in the presence of CO_2_, followed by recrystallization during cooling or depressurization. Simultaneously, the extract may act as a nucleating agent for crystal growth, promoting structural heterogeneity that affects light scattering [84] and, consequently, optical properties. Supporting this interpretation, previous observations have shown differences in crystal size and degree of crystallinity in PLA-PHB and PBS-based films before and after impregnation [17,35,85,86], as well as under different operating conditions. These structural modifications can significantly influence light–material interactions.

From an optical perspective, higher extract loadings imply a greater concentration of chromophoric compounds, enhancing radiation absorption in the UV–visible region as previously discussed. Therefore, the differences observed in the optical properties of the films are explained by the combined effect of impregnation conditions and polymer composition.

### 3.3. Influence of Polymer Blending on Impregnation Loading

**Figure 8:**
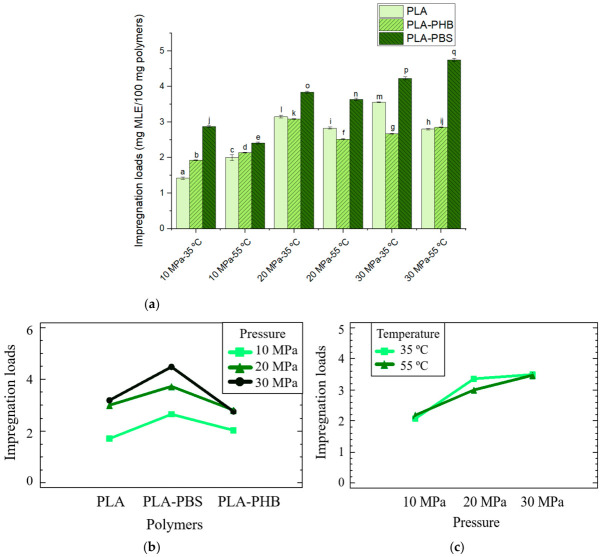
Impregnation loads of PLA, PLA-PHB and PLA-PBS films impregnated with MLE via SSI (**a**) and interaction effects of pressure–polymer formulation (**b**) and pressure–temperature (**c**). The values are the mean of at least three replicates and the error bars correspond to the confidence interval. Different letters indicate significant differences.

The impregnation loads of polymers impregnated with MLE via SSI (Figure 8a) varied depending on the polymer blend used and the conditions of the impregnation. For pure PLA, the results ranged from 1.42 to 3.56 mg MLE/100 mg polymer. The highest value was obtained by impregnating at 30 MPa and 35 °C. Generally, an increase in impregnation load was observed when the pressure was increased at 35 °C. However, at 55 °C, the impregnation load increased as pressure increased from 10 to 20 MPa and remained practically unchanged at 30 MPa. In conclusion, at higher pressures, impregnation was enhanced at low temperatures.

PLA-PBS films exhibited the highest impregnation loadings, suggesting that the incorporation of PBS into PLA enhances the impregnation of compounds from the MLE (Figure 8a). The maximum impregnation loadings were obtained at 30 MPa, indicating that, for this polymer system, increasing pressure promotes the impregnation process. However, at constant pressure, different temperature effects were observed: at 10 and 20 MPa, increasing temperature did not favor impregnation, whereas at 30 MPa, the opposite behavior was observed, with higher temperatures enhancing the impregnation loading.

The incorporation of PHB into PLA (Figure 8a) showed a tendency toward increased impregnation loading with increasing pressure at 55 °C. However, at 35 °C, the highest impregnation loading was obtained at 20 MPa (3.09 mg MLE/100 mg polymer). At 10 and 30 MPa, an increase in temperature slightly enhanced the impregnation process for this polymer system; nevertheless, at 20 MPa, increasing the temperature resulted in a 22% reduction in impregnation loading. The impregnation loading results for PLA-PHB polymers have previously been reported by León-Marcos et al. [35]. However, this study offers a novel perspective on the research by analyzing the effect of adding PHB and PBS to PLA on the impregnation loading compared to pure PLA.

The statistical analysis of the impregnation loading results revealed that polymer composition, operating pressure and their first-order interaction had a significant effect on impregnation loading (*p*-value = 0.00 in all cases) (Figure 8b and Table S6 in the Supplementary Material). In contrast, temperature did not show a statistically significant effect as an independent factor (*p*-value = 0.17); however, its interaction with pressure was found to be significant (*p*-value = 0.01) (Figure 8c and Table S6 in the Supplementary Material). When analyzing the results collectively, rather than individually for each polymer system, it can be observed that at 35 °C, impregnation loading increased with pressure up to 20 MPa, while no substantial differences were detected when pressure was further increased to 30 MPa. In contrast, at 55 °C, an increase in pressure consistently enhanced impregnation loading (Figure 8c).

As observed, the incorporation of PHB into the PLA matrix did not lead to an improvement in the impregnation loads compared to neat PLA (Figure 8b). As previously discussed, PLA is predominantly an amorphous polymer [65], whereas PHB is a semicrystalline polymer with a high degree of crystallinity (50–90%) [87]. In semicrystalline polymers, mass transfer during supercritical impregnation mainly occurs through the amorphous phase [31]. Therefore, the high crystallinity of PHB may hinder the diffusion of supercritical CO_2_ and the penetration of active compounds into the polymer matrix, which could explain the lack of improvement in impregnation loading.

In contrast, the incorporation of PBS into the PLA formulation significantly enhanced the impregnation process (Figure 8b). Several studies have reported that PBS improves the ductility of PLA [88], which may facilitate the penetration of the extract into the polymer matrix. Although PBS is also a semicrystalline polymer, it exhibits a relatively low degree of crystallinity (35–45%) [89] which can favor the diffusion of compounds during supercritical impregnation. This lower crystallinity may enhance both the penetration of supercritical CO_2_ and the extract into the PLA-PBS matrix, resulting in higher impregnation loadings. Additionally, the compounds present in MLE may interact with the polymer matrix and act as nucleating or stabilizing agents [90], further promoting this effect, particularly in PBS-containing systems.

During supercritical impregnation, an increase in operating pressure leads to a higher density of CO_2_, which enhances solute/fluid interactions and improves the solubility of the active compounds in the supercritical phase [25,62]. Consequently, a greater number of molecules become available for diffusion into the polymer structure, promoting their distribution and impregnation [91]. Furthermore, increased pressure may induce swelling of the polymer matrix, facilitating greater uptake of the active compounds [23,31,33].

However, temperature also plays a critical role in the process. Its effect is complex and depends on the polymer matrix, operating pressure and nature of the compounds present in the extract [33,60]. Although increasing temperature generally reduces CO_2_ density, it simultaneously increases the vapor pressure of the solute, which can enhance its solubility in the supercritical fluid. In addition, higher temperatures may improve diffusivity and increase the free volume of the polymer matrix, thereby favoring impregnation [92]. This behavior may explain the higher impregnation loadings observed at 30 MPa and 55 °C compared to 20 MPa and 55 °C (Figure 8c).

Conversely, an opposite effect may occur at lower temperatures. A decrease in temperature increases the density of CO_2_ and improves its transport properties [25], which can enhance the solubilization of active compounds in the supercritical phase [93]. This phenomenon may account for the behavior observed at 35 °C (Figure 8c). Fernández-Ponce et al. (2018) have previously described these competing effects, highlighting that experimental evaluation is necessary to determine the dominant mechanism in each system [25]. In this context, the higher impregnation loadings obtained at 35 °C may be attributed to the predominance of the CO_2_ density effect over the increase in solute vapor pressure. As a result, solubilization of the extract in the supercritical phase is enhanced, increasing the concentration gradient between the CO_2_ and the polymer matrix and thereby promoting more efficient impregnation.

### 3.4. Correlation Between CIELAB Color Coordinates and Impregnation Loads in PLA, PLA-PHB and PLA-PBS Films Impregnated with MLE via SSI

Cejudo-Bastante et al. established a correlation between supercritical impregnation loading of olive leaf extract and the CIELAB color parameters of the resulting films, taking advantage of the color changes induced in impregnated matrices and the chromophoric nature of the extract. This approach provided a non-destructive, rapid and efficient tool to predict the loading of impregnated PET/PP films [36]. This methodology was later successfully applied by Machado et al. in the evaluation of supercritical fluid impregnation scale-up processes [94].

**Figure 9:**
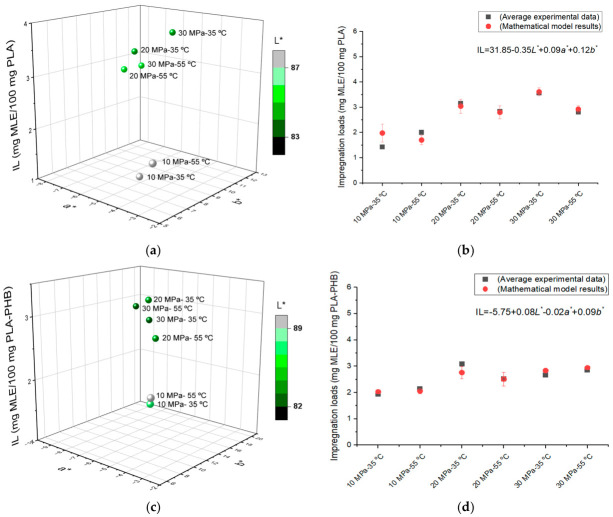

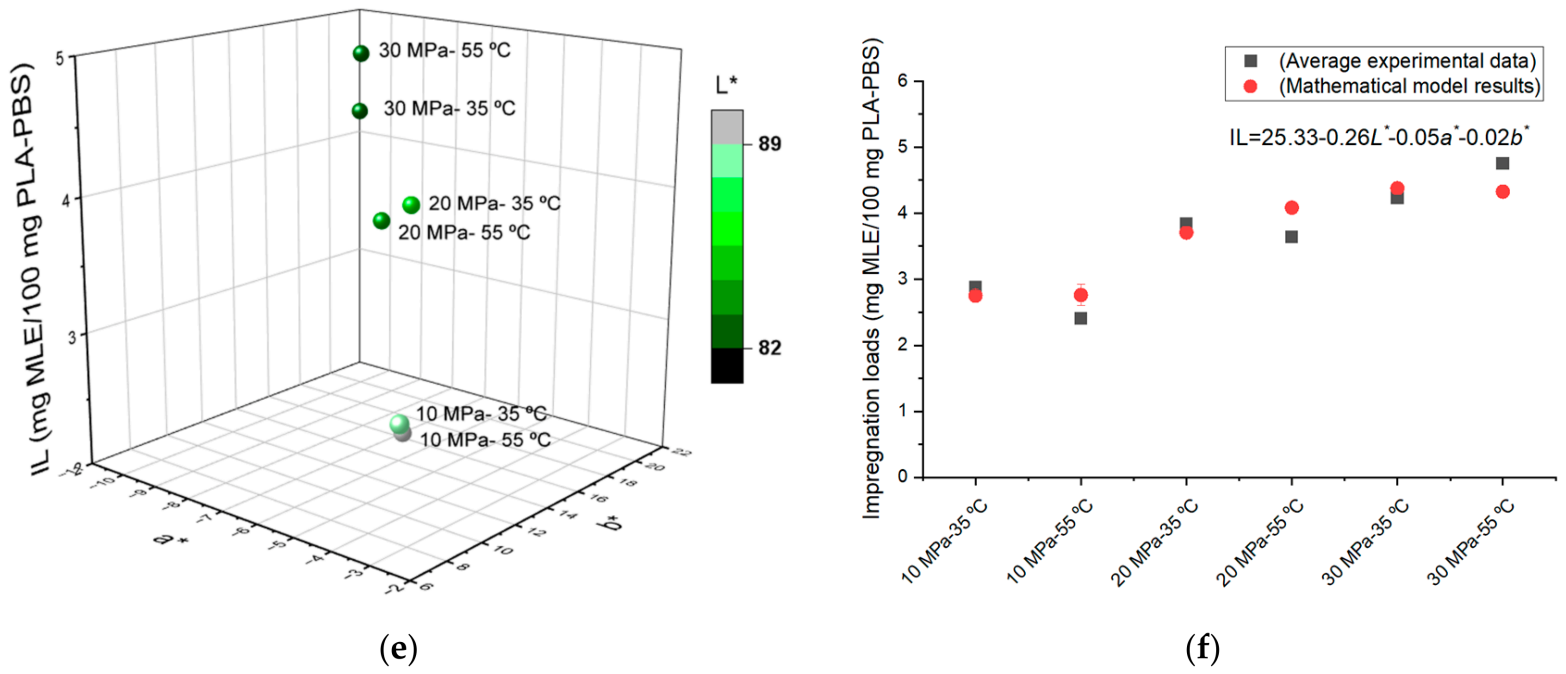
4D plots showing the relationship between impregnation loading and color properties of PLA (**a**), PLA-PHB (**c**) and PLA-PBS (**e**) films impregnated with MLE via SSI and comparison between experimental and predicted impregnation loading values obtained from the multiple linear regression model for PLA (**b**), PLA-PHB (**d**) and PLA-PBS (**f**) films.

Based on these advantages, the present study aims to establish a correlation between the CIELAB coordinates of PLA, PLA-PHB and PLA-PBS films impregnated with mango leaf extract via SSI. In this context, Figure 9a,c,e illustrate a four-dimensional relationship between colorimetric properties and impregnation loading for each polymer system and impregnation condition. These figures complement the previously discussed results, clearly showing that the impregnation loading of MLE was closely related to variations in the CIELAB color coordinates of the films for all evaluated polymer systems. In general, samples with higher impregnation loading exhibited more pronounced changes in their colorimetric properties, particularly under higher-pressure conditions, where the highest loading values were obtained. These findings support the use of CIELAB color parameters as potential indicators for estimating impregnation loading in PLA-based films processed via SSI.

In order to confirm a correlation between CIELAB parameters and impregnation loading, a multivariate analysis was performed. The multiple linear regression models developed for IL exhibited strong overall statistical significance across all polymer systems studied (*p*-value = 0.00) (Table 4), indicating that the combination of L*, a* and b* variables significantly explains the variability of the response. However, individual coefficient analysis revealed that, for PLA and PLA-PBS, only the L* parameter was statistically significant (*p*-value = 0.00) (Table 5), while for PLA-PHB the L* and a* parameters were statistically significant (*p*-value = 0.00 and *p*-value = 0.01) (Table 5).

This behavior may be attributed to the inherent correlation among the CIELAB parameters. In the CIELAB color space, L*, a* and b* represent interdependent components of color perception. Additionally, the impregnation process simultaneously affects all three parameters, indicating that these variables do not vary independently but rather as a response to the same physical phenomenon. Therefore, although individual variables may not appear significant, their combined contribution remains relevant—as reflected in the overall model significance. It is important to note that this does not invalidate the models, as their primary objective is to make predictions. The models allow estimation of impregnation loading based on the combined colorimetric parameters. Considering their strong overall performance, all variables were retained in the models, focusing on predictive capability over individual coefficient interpretation.

The developed models exhibited high coefficients of determination (R^2^ ranging from 86.37% to 87.76%) (Table 4 and Table 6), indicating a good predictive capacity of impregnation loading from L*, a* and b* parameters. Differences observed among the equations suggest that the relationship between colorimetric properties and impregnation loading is dependent on the polymer matrix.

Figure 9b,d,f show the comparison between experimental and predicted impregnation loading values obtained from the regression models. A high level of agreement was observed, confirming the ability of the models to accurately reproduce experimental behavior without significant deviations.

In summary, this study establishes a correlation between impregnation loading of mango leaf extract in PLA, PLA-PHB and PLA-PBS films processed via SSI and their colorimetric properties, providing a non-destructive, rapid and efficient predictive tool. Nowadays, the determination of total impregnation loading in polymeric matrices by supercritical impregnation presents certain challenges, particularly due to the typically low impregnation levels achieved [36]. Several methods have been reported for its quantification, including gravimetric determination based on the weight difference before and after the impregnation process [95]. However, this technique is not suitable when low impregnation levels are obtained [36].

Alternative approaches involve dissolving the impregnated polymer in an appropriate solvent and determining impregnation loading through the absorbance of the incorporated compounds. Nevertheless, these methodologies often require the use of environmentally less favorable solvents, such as dichloromethane or chloroform, due to the limited solubility of many polymers in greener solvents [35,71,96]. This approach was employed in the present study to quantify the impregnation loading of samples processed via SSI. Other analytical techniques, including UV spectroscopy and liquid or gas chromatography, have also been used to determine impregnation loading after extracting the active compounds from the polymer matrix [97]. However, incomplete extraction may occur when the interactions between the impregnated compounds and the polymer matrix are stronger than those between the compounds and the extraction medium, or when the extraction medium does not adequately diffuse into the polymer structure [36]. In addition, chromatographic techniques generally require analytical standards and involve more complex and time-consuming procedures.

In this context, the correlations developed in the present work represent a promising alternative for the indirect estimation of impregnation loading through colorimetric analysis. However, it should be noted that the films evaluated in this study were prepared by solvent casting and impregnated via SSI. Therefore, these correlations should not be directly extrapolated to films produced or impregnated using different techniques, or to systems containing different plasticizers or additives, since these factors may influence film crystallinity, active compound distribution and consequently the optical and colorimetric responses of the materials. For example, Cejudo-Bastante et al. reported differences in the color properties of nanocellulose films impregnated with mango leaf extract using supercritical impregnation and solvent casting techniques [26]. Accordingly, the application of these predictive models requires prior validation under the specific processing and formulation conditions of interest.

### 3.5. Release Study of MLE Impregnated in PLA, PLA-PHB and PLA-PBS Films via SSI

**Figure 10:**
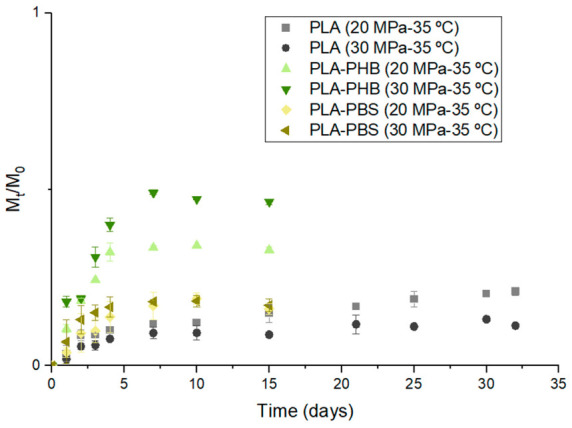
Release kinetics in ethanol 50% of MLE from PLA, PLA-PHB and PLA-PBS films impregnated via SSI at 20 and 30 MPa and 35 °C.

In the release study of active compounds from the three evaluated polymer matrices, an initial rapid release stage was observed during the first 24 h of contact between the films and the food simulant. This was followed by a reduced extraction rate, which, in the case of PLA, extended up to approximately 30 days (Figure 10). Similar prolonged release behaviors of phenolic antioxidants from PLA films in ethanol 50% have been reported in the literature [98].

During the release process, several factors play a key role, including the extraction capacity of the food simulant, its ability to diffuse into the polymer matrix and dissolve the impregnated compounds [99], as well as the interactions between the polymer matrix and the active compounds [98]. When the interactions between the matrix and the active compounds are stronger than the affinity of the compounds for the extraction medium, the release is hindered, resulting in lower extraction levels.

The interaction between phenolic compounds and PLA has been widely documented [33,100]. These interactions are mainly governed by hydrogen bonding between the hydroxyl groups of phenolic compounds and the carbonyl groups of PLA, forming –C=O···HO- interactions that can also reduce polymer chain mobility [100]. A limited release of incorporated phenolic acids from PLA-based films has been reported, indicating that diffusion through the polymer network is slow and highly dependent on both the polymer structure and polymer–compound interactions [100]. This behavior is consistent with the results obtained from the fitting and determination of the Peleg model parameters (Table 7), where PLA films exhibited the lowest initial release rate (1/k_1_) among all samples. Furthermore, they reached the lowest equilibrium release levels, with the ratio of active compounds released at equilibrium (M_∞_/M_o_) ranging between 15 and 24%.

On the other hand, in PLA-PHB blend films, a higher release of MLE from the polymer matrix was observed (Figure 10). Additionally, based on the good fitting of the Peleg model (Table 7), PLA–PHB exhibited the highest initial release rate (1/k_1_) among the studied polymers, which contributed to the greater amount of compounds released from this system. Similarly, Arrieta M. P. et al. reported that the incorporation of PHB into PLA matrices led to a slight increase and faster release of catechin compared to neat PLA formulations. They attributed this behavior to a higher affinity of the D1 food simulant for PLA-PHB blends, which enhanced the release rate of the active compound over time [18].

Nevertheless, the release of impregnated compounds remained partial, indicating that the interactions between the active compounds and the polymer matrix were stronger than the extraction capacity of the food simulant. In agreement with this, Hernández-García E. et al. also reported limited release of phenolic acids in food simulants from PLA–poly(3-hydroxybutyrate-co-3-hydroxyvalerate) (PHBV) blend films [28].

In this polymeric system (PLA-PHB), no differences were observed in the initial release rate (1/k_1_) when varying the impregnation conditions. However, higher released concentrations were achieved for samples impregnated at 30 MPa and 35 °C. The increase in pressure during impregnation may have induced a rearrangement of the polymer chains, facilitating the diffusion of the food simulant into the matrix and, consequently, enhancing the release of MLE. Furthermore, lower impregnation temperatures likely minimize thermal deformation or structural alterations within the PLA-PHB matrix, thereby facilitating the diffusion of active compounds into the release medium. This observation aligns with León-Marcos et al., who reported enhanced release kinetics at lower temperatures coupled with higher impregnation pressures [35].

In contrast, the release study of MLE impregnated in PLA-PBS blend films showed low release rates despite exhibiting the highest impregnation loadings. Equilibrium was reached after approximately 7 days (Figure 10), with partial release values (M_∞_/M_o_) ranging between 19 and 20% (Table 7). In this case, the best fit was obtained using the first-order model. Łukasz Łopusiewicz et al., in a study on quercetin-loaded PBS films, also reported partial release of the compound in this food simulant, with the highest released concentrations observed at the longest evaluated times [72]. Strong interactions between phenolic compounds and PBS matrices have also been reported, mainly attributed to hydrogen bonding and van der Waals interactions between the hydroxyl groups of the phenolic compounds and the ester carbonyl groups of PBS [56,101]. These interactions may explain both the high impregnation loadings and the partial release observed in PLA-PBS systems.

Finally, the parameters obtained from the Korsmeyer–Peppas model showed diffusion exponent (n) values lower than 0.5 in all cases, indicating that quasi-Fickian diffusion mechanisms [45,47] govern the release of the impregnated compounds in PLA, PLA-PHB and PLA-PBS films.

## 4. Conclusions

This study demonstrated that both polymer composition and supercritical impregnation conditions significantly influenced the optical, chromatic, impregnation and release properties of PLA-based films.

The incorporation of PHB and PBS into the PLA matrix modified the visual appearance of the non-impregnated films, particularly by increasing the b* parameter. Following impregnation, all films showed clear chromatic modifications, characterized by lower lightness and more marked yellowish-green tones. In addition, the presence of PHB and PBS significantly increased film opacity and reduced transparency, while simultaneously improving the UV-light barrier properties of the developed materials, which is highly relevant for active food-packaging applications.

On the other hand, the addition of PBS to PLA markedly enhanced the incorporation of MLE, leading to the highest impregnation loading values among all the formulations studied (2.41–4.75 mg MLE/100 mg polymer). In contrast, the incorporation of PHB did not improve impregnation loading compared with neat PLA. A strong relationship was established between CIELAB color coordinates and impregnation loading and robust multiple linear regression models were developed for the three polymer systems, all showing coefficients of determination above 85%, thus providing a rapid and non-destructive predictive tool.

Finally, all films exhibited partial release of the impregnated compounds, although with clearly differentiated kinetic profiles depending on the polymer matrix. PLA films showed the slowest release, whereas PLA-PHB films presented the highest proportion of released compounds. The release process was predominantly governed by quasi-Fickian diffusion mechanisms in all systems.

## Figures and Tables

**Table 1:** Thickness of the PLA, PLA-PHB and PLA-PBS films before and after MLE impregnation via SSI.

	Impregnation Conditions	Thickness (mm)
PLA	Non-Impregnated	0.06 ± 0.001 ^a^
	10 MPa-35 °C	0.07 ± 0.003 ^e^
	10 MPa-55 °C	0.10 ± 0.001 ^ij^
	20 MPa-35 °C	0.10 ± 0.010 ^hi^
	20 MPa-55 °C	0.09 ± 0.004 ^gh^
	30 MPa-35 °C	0.10 ± 0.001 ^ij^
	30 MPa-55 °C	0.10 ± 0.001 ^ij^
PLA-PHB	Non-Impregnated	0.06 ± 0.001 ^ab^
	10 MPa-35 °C	0.08 ± 0.010 ^efg^
	10 MPa-55 °C	0.08 ± 0.010 ^efg^
	20 MPa-35 °C	0.08 ± 0.003 ^ef^
	20 MPa-55 °C	0.07 ± 0.002 ^e^
	30 MPa-35 °C	0.09 ± 0.010 ^gh^
	30 MPa-55 °C	0.10 ± 0.010 ^hi^
PLA-PBS	Non-Impregnated	0.06 ± 0.001 ^a^
	10 MPa-35 °C	0.07 ± 0.004 ^de^
	10 MPa-55 °C	0.09 ± 0.010 ^g^
	20 MPa-35 °C	0.07 ± 0.010 ^bc^
	20 MPa-55 °C	0.07 ± 0.001 ^bcd^
	30 MPa-35 °C	0.06 ± 0.001 ^ab^
	30 MPa-55 °C	0.07 ± 0.001 ^cd^

Note: The values are the mean of at least three replicates and the error bars correspond to the confidence interval. Different letters indicate significant differences among the samples in the same column.

**Table 2:** CIELAB coordinates and Δ*E* values of PLA, PLA-PHB and PLA-PBS films before and after MLE impregnation via SSI.

	Impregnation Conditions	L*	a*	b*	Δ*E* Values (Respect to Non-Impregnated Polymer)
PLA	Non-Impregnated	89.82 ± 0.06 ^j^	−1.40 ± 0.11 ^j^	1.64 ± 0.60 ^a^	
	10 MPa-35 °C	87.67 ± 0.25 ^h^	−3.97 ± 0.69 ^g^	6.47 ± 1.53 ^d^	6.08 ± 1.54 ^b^
	10 MPa-55 °C	88.07 ± 0.10 ^hi^	−2.39 ± 0.10 ^i^	5.09 ± 0.76 ^c^	4.21 ± 0.73 ^a^
	20 MPa-35 °C	84.68 ± 0.30 ^e^	−7.73 ± 0.23 ^d^	11.42 ± 1.76 ^g^	12.94 ± 1.16 ^f^
	20 MPa-55 °C	85.52 ± 0.47 ^f^	−7.71 ± 0.38 ^d^	10.54 ± 0.34 ^f^	11.9 ± 0.27 ^e^
	30 MPa-35 °C	84.41 ± 0.11 ^de^	−6.35 ± 0.05 ^e^	12.60 ± 0.54 ^h^	13.39 ± 0.51 ^f^
	30 MPa-55 °C	85.81 ± 0.27 ^f^	−7.81 ± 0.02 ^d^	12.12 ± 0.02 ^gh^	13.11 ± 0.07 ^f^
PLA-PHB	Non-Impregnated	90.85 ± 0.34 ^j^	−1.39 ± 0.05 ^j^	2.61 ± 0.32 ^a^	
	10 MPa-35 °C	86.68 ± 0.28 ^g^	−3.76 ± 0.58 ^g^	7.88 ± 0.58 ^e^	7.13 ± 0.76 ^c^
	10 MPa-55 °C	89.49 ± 0.17 ^j^	−3.00 ± 0.27 ^h^	6.13 ± 1.04 ^d^	4.11 ± 1.03 ^a^
	20 MPa-35 °C	83.44 ± 2.53 ^c^	−7.53 ± 0.10 ^d^	16.55 ± 0.29 ^k^	16.97 ± 0.87 ^h^
	20 MPa-55 °C	84.00 ± 1.97 ^cd^	−6.62 ± 0.35 ^e^	15.55 ± 0.98 ^i^	15.58 ± 0.02 ^g^
	30 MPa-35 °C	82.24 ± 0.49 ^a^	−8.33 ± 0.02 ^c^	18.60 ± 0.45 ^L^	19.44 ± 0.15 ^i^
	30 MPa-55 °C	82.79 ± 0.15 ^ab^	−9.46 ± 0.21 ^b^	19.01 ± 0.23 ^lm^	19.98 ± 0.09 ^i^
PLA-PBS	Non-Impregnated	90.03 ± 0.01 ^j^	−1.39 ± 0.03 ^j^	1.74 ± 0.02 ^b^	
	10 MPa-35 °C	88.47 ± 0.05 ^i^	−3.07 ± 0.04 ^h^	7.71 ± 0.03 ^e^	6.38 ± 0.03 ^bc^
	10 MPa-55 °C	89.52 ± 1.19 ^j^	−5.00 ± 1.34 ^f^	12.09 ± 1.44 ^gh^	10.98 ± 1.90 ^d^
	20 MPa-35 °C	84.93 ± 0.05 ^e^	−6.50 ± 0.03 ^e^	15.78 ± 0.09 ^ij^	15.78 ± 0.10 ^g^
	20 MPa-55 °C	83.65 ± 0.10 ^c^	−7.63 ± 0.07 ^d^	16.39 ± 0.10 ^jk^	17.15 ± 0.10 ^h^
	30 MPa-35 °C	82.79 ± 0.02 ^ab^	−10.38 ± 0.77 ^a^	20.73 ± 0.01 ^n^	22.22 ± 0.30 ^k^
	30 MPa-55 °C	82.86 ± 0.05 ^b^	−9.75 ± 0.29 ^b^	19.51 ±1.19 ^m^	20.91 ± 1.10 ^j^

Note: The values are the mean of at least three replicates and the error bars correspond to the confidence interval. Different letters indicate significant differences among the samples in the same column.

**Table 3:** The chroma (C*ab), hue angle (h*ab) value, whiteness index (WI) and yellowness index (YI) for PLA, PLA-PHB and PLA-PBS films before and after MLE impregnation via SSI.

	Impregnation Conditions	C*ab	h*ab	WI	YI
PLA	Non-Impregnated	2.01 ± 0.01 ^a^	136.28 ± 0.36 ^L^	89.65 ± 0.06 ^m^	1.59 ± 0.00 ^b^
	10 MPa-35 °C	7.60 ± 1.54 ^e^	121.70 ± 5.35 ^h^	85.50 ± 1.00 ^k^	1.63 ± 0.01 ^d^
	10 MPa-55 °C	5.62 ± 0.73 ^c^	115.22 ± 2.60 ^def^	86.81 ± 0.39 ^L^	1.62 ± 0.00 ^cd^
	20 MPa-35 °C	13.81 ± 1.32 ^gh^	124.21 ± 5.00 ^ij^	79.37 ± 0.75 ^fg^	1.69 ± 0.01 ^g^
	20 MPa-55 °C	13.05 ± 0.22 ^g^	126.20 ± 2.02 ^j^	80.51 ± 0.40 ^h^	1.67 ± 0.01 ^f^
	30 MPa-35 °C	14.11 ± 0.50 ^h^	116.77 ± 0.83 ^fg^	78.97 ± 0.42 ^f^	1.69 ± 0.00 ^gh^
	30 MPa-55 °C	14.42 ± 0.01 ^h^	122.80 ± 0.09 ^hi^	79.76 ± 0.18 ^g^	1.66 ± 0.01 ^f^
PLA-PHB	Non-Impregnated	2.86 ± 0.19 ^ab^	119.25 ± 3.19 ^g^	90.28 ± 0.25 ^n^	1.57 ± 0.01 ^a^
	10 MPa-35 °C	8.93 ± 0.47 ^f^	115.51 ± 1.11 ^def^	83.47 ± 1.55 ^j^	1.66 ± 0.03 ^e^
	10 MPa-55 °C	7.07 ± 0.68 ^d^	115.80 ± 1.17 ^def^	87.32 ± 0.45 ^L^	1.60 ± 0.00 ^b^
	20 MPa-35 °C	18.11 ± 0.19 ^j^	114.46 ± 0.02 ^bcde^	75.05 ± 0.97 ^d^	1.72 ± 0.03 ^j^
	20 MPa-55 °C	16.90 ± 0.66 ^i^	113.07 ± 0.13 ^abc^	76.72 ± 0.40 ^e^	1.70 ± 0.03 ^hi^
	30 MPa-35 °C	20.38 ± 0.28 ^k^	114.33 ± 0.49 ^bcd^	73.06 ± 0.22 ^c^	1.73 ± 0.01 ^k^
	30 MPa-55 °C	21.22 ± 0.07 ^L^	116.44 ± 0.53 ^efg^	72.53 ± 0.35 ^bc^	1.73 ± 0.01 ^k^
PLA-PBS	Non-Impregnated	2.23 ± 0.03 ^b^	128.57 ± 0.26 ^k^	89.79 ± 0.01 ^mn^	1.59 ± 0.00 ^b^
	10 MPa-35 °C	8.29 ± 0.03 ^ef^	111.72 ± 0.17 ^a^	85.80 ± 0.06 ^k^	1.61 ± 0.00 ^c^
	10 MPa-55 °C	13.09 ± 1.84 ^g^	112.38 ± 3.04 ^ab^	83.24 ± 2.18 ^i^	1.60 ± 0.02 ^b^
	20 MPa-35 °C	17.06 ± 0.09 ^i^	112.39 ± 0.04 ^ab^	77.23 ± 0.10 ^e^	1.68 ± 0.00 ^g^
	20 MPa-55 °C	18.08 ± 0.12 ^j^	114.97 ± 0.09 ^cdef^	75.62 ± 0.16 ^d^	1.71 ± 0.00 ^ij^
	30 MPa-35 °C	23.18 ± 0.35 ^m^	116.59 ± 1.71 ^fg^	71.12 ± 0.27 ^a^	1.73 ± 0.01 ^k^
	30 MPa-55 °C	21.81 ± 1.19 ^L^	116.56 ± 0.73 ^fg^	72.25 ± 0.90 ^b^	1.72 ± 0.00 ^k^

Note: The values are the mean of at least three replicates and the error bars correspond to the confidence interval. Different letters indicate significant differences among the samples in the same column.

**Table 4:** Analysis of Variance for correlation between CIELAB color coordinates and impregnation loads in PLA, PLA-PHB and PLA-PBS films impregnated with MLE via SSI.

	**Source**	**Sum of Squares**	**Df**	**Mean Square**	**F-Ratio**	***p*-Value**
PLA	Model	7.97	3	2.66	30.48	0.00
	Residual	1.22	14	0.09		
	Total (Corr.)	9.19	17			
	R^2^ = 86.72%					
PLA-PHB	**Source**	**Sum of Squares**	**Df**	**Mean Square**	**F-Ratio**	***p*-Value**
	Model	2.19	3	0.73	15.09	0.00
	Residual	0.68	14	0.05		
	Total (Corr.)	2.87	17			
	R^2^ = 86.37%					
PLA-PBS	**Source**	**Sum of Squares**	**Df**	**Mean Square**	**F-Ratio**	***p*-Value**
	Model	9.83	3	3.28	33.47	0.00
	Residual	1.37	14	0.09		
	Total (Corr.)	11.19	17			
	R^2^ = 87.76%					

**Table 5:** Analysis of Variance for variables in the order fitter in the correlation between CIELAB color coordinates and impregnation loads in PLA, PLA-PHB and PLA-PBS films impregnated with MLE via SSI.

	**Source**	**Sum of Squares**	**Df**	**Mean Square**	**F-Ratio**	***p*-Value**
PLA	L*	7.71	1	7.71	88.44	0.00
	a*	0.01	1	0.01	0.06	0.81
	b*	0.26	1	0.26	2.94	0.11
	Model	7.97	3			
PLA-PHB	**Source**	**Sum of Squares**	**Df**	**Mean Square**	**F-Ratio**	***p*-Value**
	L*	1.69	1	1.69	34.84	0.00
	a*	0.43	1	0.43	8.84	0.01
	b*	0.08	1	0.08	1.59	0.23
	Model	2.19	3			
PLA-PBS	**Source**	**Sum of Squares**	**Df**	**Mean Square**	**F-Ratio**	***p*-Value**
	L*	9.81	1	9.81	100.25	0.00
	a*	0.01	1	0.01	0.13	0.72
	b*	0.00	1	0.00	0.03	0.86
	Model	9.82	3			

**Table 6:** Multiple linear regression equations describing the relationship between CIELAB color coordinates and impregnation loading in PLA, PLA-PHB and PLA-PBS films impregnated with MLE via SSI.

Polymers	Multiple Linear Regression Equations	R^2^	
PLA	$IL=31.85-0.35L^{*}+0.09a^{*}+0.12b^{*}$	86.72	(19)
PLA-PHB	$IL=-5.75+0.08L^{*}-0.02a^{*}+0.09b^{*}$	86.37	(20)
PLA-PBS	$IL=25.33-0.26L^{*}-0.05a^{*}-0.02b^{*}$	87.76	(21)

**Table 7:** Fitting results of release kinetics models of MLE from PLA, PLA-PHB and PLA-PBS films impregnated via SSI at 20 and 30 MPa and 35 °C and released in ethanol 50%.

	PLA		PLA-PHB		PLA-PBS	
	20 MPa 35 °C	30 MPa 35 °C	20 MPa 35 °C	30 MPa 35 °C	20 MPa 35 °C	30 MPa 35 °C
Zero-order						
k	*	*	*	*	*	*
R^2^	*	*	*	*	*	*
First-order						
k	0.003	0.004	0.008	0.006	0.005	0.009
R_0_	0.19	0.11	0.33	0.46	0.18	0.18
R^2^	0.90	0.93	0.94	0.93	0.96	0.99
Peleg model						
1/k_1_	0.003	0.002	0.02	0.02	0.004	0.008
k_2_	4.25	6.79	3.13	2.15	5.08	5.38
R^2^	0.97	0.97	0.96	0.95	0.91	0.98
M_∞_/M_0_ (%)	24	15	32	46	20	19
Power Law/Korsmeyer–Peppas model						
k	0.01	0.01	0.06	0.06	0.015	0.04
n	0.38	0.32	0.26	0.30	0.38	0.23
R^2^	0.97	0.92	0.80	0.82	0.86	0.88
Higuchi						
k	0.01	0.003	0.01	0.02	0.007	0.007
R^2^	0.95	0.82	0.66	0.74	0.84	0.64
Avrami						
k	0.01	0.01	0.05	0.05	0.01	0.04
n	0.41	0.33	0.32	0.40	0.41	0.25
R^2^	0.96	0.92	0.81	0.84	0.86	0.88

* indicates poor fit to the model (R^2^ ≤ 0.50).

## Data Availability

The original contributions presented in this study are included in the article/Supplementary Material. Further inquiries can be directed to the corresponding author.

## Supplementary Materials

The following supporting information can be downloaded at: https://www.mdpi.com/article/10.3390/polym18111377/s1, Table S1: Analysis of Variance for *L** parameter of PLA, PLA-PHB and PLA-PBS films impregnated with mango leaf extract (MLE) via SSI; Table S2: Analysis of Variance for *a** parameter of PLA, PLA-PHB and PLA-PBS films impregnated with MLE via SSI; Table S3: Analysis of Variance for *b** parameter of PLA, PLA-PHB and PLA-PBS films impregnated with MLE via SSI; Table S4: Analysis of Variance for opacity of PLA, PLA-PHB and PLA-PBS films impregnated with MLE via SSI; Table S5: Analysis of Variance for transparency of PLA, PLA-PHB and PLA-PBS films impregnated with MLE via SSI; Table S6: Analysis of Variance for impregnation loads of PLA, PLA-PHB and PLA-PBS films impregnated with MLE via SSI.
