# Supplementary material for: Supercritical Solvent Impregnation of Poly(lactic acid) (PLA)-Based Films: Effect of Poly(3-hydroxybutyrate) (PHB) and Poly(butylene succinate) (PBS) on Loading Capacity, Optical Properties and Release Kinetics of Mango Leaf Extract
Source: Polymers (Basel). 2026 Jun 1;18(11):1377. doi: 10.3390/polym18111377 (PMC13259300; doi:10.3390/polym18111377)
Supplement: Supplementary file 1 [file polymers-18-01377-s001.zip › polymers-4314541-supplementary.pdf]

Supercritical solvent impregnation of poly(lactic acid) (PLA)-based films: effect of poly(3-hydroxybutyrate) (PHB) and poly(butylene succinate) (PBS) on loading capacity, optical properties and release kinetics of mango leaves extract

Ludisbel León-Marcos[a], Antonio Montes[a], Diego Valor[a], Ignacio García-Casas[a] and Clara Pereyra[a].*

[a]Department of Chemical Engineering and Food Technology, Faculty of Sciences, University of Cadiz, International Excellence Agrifood Campus (CeiA3), Wine and Agrifood Research Institute (IVAGRO), 11510 Puerto Real (Cadiz), Spain.

*Correspondence: antonio.montes@uca.es

Table S1. Analysis of Variance for *L** parameter of PLA, PLA-PHB and PLA-PBS films impregnated with mango leaf extract (MLE) via SSI.

Source	*Sum of Squares*	*Df*	*Mean Square*	*F-Ratio*	*P-Value*
**MAIN EFFECTS**					
**A:Polymers**	14.11	2	7.056	26.66	0.00
**B:Pressure**	238.53	2	119.27	450.60	0.00
**C:Temperature**	6.86	1	6.86	25.90	0.00
**INTERACTIONS**					
**AB**	20.01	4	5.00	18.90	0.00
**AC**	4.38	2	2.19	8.27	0.00
**BC**	4.28	2	2.14	8.09	0.00
**RESIDUAL**	10.59	40	0.26		
**TOTAL (CORRECTED)**	298.76	53			

Table S2. Analysis of Variance for *a** parameter of PLA, PLA-PHB and PLA-PBS films impregnated with MLE via SSI.

Source	*Sum of Squares*	*Df*	*Mean Square*	*F-Ratio*	*P-Value*
**MAIN EFFECTS**					
**A:Polymers**	11.48	2	5.74	13.09	0.00
**B:Pressure**	262.74	2	131.37	299.61	0.00
**C:Temperature**	0.78	1	0.78	1.78	0.19
**INTERACTIONS**					
**AB**	20.73	4	5.18	11.82	0.00
**AC**	2.37	2	1.18	2.70	0.08
**BC**	1.17	2	0.58	1.33	0.28
**RESIDUAL**	17.54	40	0.44		
**TOTAL (CORRECTED)**	316.80	53			

Table S3. Analysis of Variance for *b** parameter of PLA, PLA-PHB and PLA-PBS films impregnated with MLE via SSI.

Source	*Sum of Squares*	*Df*	*Mean Square*	*F-Ratio*	*P-Value*
**MAIN EFFECTS**					
**A:Polymers**	320.61	2	160.31	186.46	0.00
**B:Pressure**	882.4	2	441.2	513.18	0.00
**C:Temperature**	0.13	1	0.13	0.15	0.69
**INTERACTIONS**					
**AB**	47.98	4	11.99	13.95	0.00
**AC**	12.47	2	6.24	7.25	0.00
**BC**	2.89	2	1.45	1.68	0.19
**RESIDUAL**	34.39	40	0.86		
**TOTAL (CORRECTED)**	1300.88	53			

Table S4. Analysis of Variance for Opacity of PLA, PLA-PHB and PLA-PBS films impregnated with MLE via SSI.

Source	*Sum of Squares*	*Df*	*Mean Square*	*F-Ratio*	*P-Value*
**MAIN EFFECTS**					
**A:Polymers**	2278.45	2	1139.23	782.19	0.00
**B:Pressure**	8.58	2	4.29	2.94	0.06
**C:Temperature**	15.38	1	15.38	10.56	0.00
**INTERACTIONS**					
**AB**	107.71	4	26.93	18.49	0.00
**AC**	9.26	2	4.63	3.18	0.05
**BC**	42.74	2	21.37	14.67	0.00
**RESIDUAL**	58.26	40	1.46		
**TOTAL (CORRECTED)**	2520.38	53			

Table S5. Analysis of Variance for Transparency of PLA, PLA-PHB and PLA-PBS films impregnated with MLE via SSI.

Source	*Sum of Squares*	*Df*	*Mean Square*	*F-Ratio*	*P-Value*
**MAIN EFFECTS**					
**A:Polymers**	209.32	2	104.66	26.87	0.00
**B:Pressure**	149,41	2	74,71	19,18	0,00
**C:Temperature**	204,79	1	204,79	52,58	0,00
**INTERACTIONS**					
**AB**	662.42	4	165.61	42.52	0.00
**AC**	10.58	2	5.29	1.36	0.27
**BC**	68.91	2	34.46	8.85	0.00
**RESIDUAL**	155.81	40	3.89		
**TOTAL (CORRECTED)**	1461.25	53			

Table S6. Analysis of Variance for Impregnation loads of PLA, PLA-PHB and PLA-PBS films impregnated with MLE via SSI.

Source	*Sum of Squares*	*Df*	*Mean Square*	*F-Ratio*	*P-Value*
**MAIN EFFECTS**					
**A:Polymers**	13.08	2	6.54	117.51	0.00
**B:Pressure**	18.11	2	9.05	162.70	0.00
**C:Temperature**	0.11	1	0.11	1.97	0.17
**INTERACTIONS**					
**AB**	2.23	4	0.56	10.02	0.00
**AC**	0.04	2	0.02	0.33	0.72
**BC**	0.54	2	0.27	4.88	0.01
**RESIDUAL**	2.23	40	0.06		
**TOTAL (CORRECTED)**	36.33	53			
